# Expression profile and gap-junctional transfer of microRNAs in the bovine cumulus-oocyte complex

**DOI:** 10.3389/fcell.2024.1404675

**Published:** 2024-07-11

**Authors:** R. Six, C. Benedetti, Y. Fan, X. Guan, Y. Gansemans, Mohamed Hedia, O. Bogado Pascottini, K. C. Pavani, F. Van Nieuwerburgh, D. Deforce, K. Smits, A. Van Soom, L. Peelman

**Affiliations:** ^1^ Department of Veterinary and Biosciences, Ghent University, Merelbeke, Belgium; ^2^ Department of Internal Medicine, Reproduction and Population Medicine, Ghent University, Merelbeke, Belgium; ^3^ Laboratory of Pharmaceutical Biotechnology, Faculty of Pharmaceutical Sciences, Ghent University, Ghent, Belgium; ^4^ Theriogenology Department, Faculty of Veterinary Medicine, Cairo University, Giza, Egypt

**Keywords:** miRNA, gap junction, bovine oocyte, intercellular communication, maturation, cumulus cells

## Abstract

MicroRNAs (miRNA) are important regulators of oocyte maturation, playing a key role in modulating gene expression both in a temporal- and spatial-specific manner. These small non-coding RNAs are involved in important processes during oocyte maturation, acting as messengers between the oocyte and its surrounding cumulus cells. Despite its significance, the bidirectional communication mechanism is still unknown. To test miRNA communication between oocyte and surrounding cumulus cells through the gap junctions the gap junctions were either blocked with carbenoxolone or not. MiRNA sequencing of oocytes at 1, 6, and 22 h of *in vitro* maturation was then performed. Among the differentially expressed miRNAs, bta-miR-21-5p, a regulator of cumulus cell viability and oocyte maturation, was the only previously known miRNA. Furthermore, by labeling a bta-miR-21-5p mimic with FAM, crossing of this miRNA through the gap junctions within the cumulus-oocyte complex could be visualized and internalization in the oocyte was confirmed by RT-qPCR. In conclusion, this study provides, for the first time, evidence that miRNA communication within the bovine cumulus-oocyte complex is enabled through the gap junctional network.

## 1 Introduction

MicroRNAs (miRNAs) are small non-coding RNAs involved in post-transcriptional regulation of genes. They bind to miRNA response elements that are mostly situated in the 3′ untranslated region of the target messenger RNAs (mRNAs). This binding leads to either mRNA degradation or translational repression. Many miRNAs are highly conserved across evolutionarily distant organisms and involved in various biological processes, including cell differentiation, proliferation, and apoptosis (Bartel, 2009). Based on these characteristics, miRNAs are suggested to play an important role in fundamental evolutionally conserved mechanisms, including oogenesis (Tesfaye et al., 2018).

During oogenesis, the maternal mRNA transcription is halted from the germinal vesicle break down until after fertilization when the embryonic genome is activated (Hale et al., 2020). This implies that finalization of the second meiosis and fertilization proceeds with the stored mRNAs and proteins from the early-stage oocyte. Therefore, it is suggested that post-transcriptional gene regulation is crucial during oocyte maturation. In consequence, as a class of the most important post-transcriptional gene regulators, miRNAs are probably also important during oocyte maturation (Abd El Naby et al., 2013).

Since miRNAs target both autocrine and paracrine mRNAs, they can be transported to surrounding cells (Sohel et al., 2013). This transport can occur either directly, using a network of gap junctions (GJ) (juxtacrine) or from a distance (autocrine, endocrine, or paracrine). The latter, which is defined as communication between nearby cells by secreting factors in the extracellular environment, can occur via extracellular vesicles (EVs) or vesicle-free, bounded to a protein (often AGO2) (Xie et al., 2023). Since micro-vesicle-based genetic communication is less efficient due to unavoidable dilutions in the extracellular environment, it is suggested that important cellular processes require GJ-dependent communication (Brink et al., 2012).

Within the ovarian follicle, communication occurs bidirectionally between the oocyte and the cumulus cells (Buccione et al., 1990). These cumulus cells (often called corona radiata cells) are the inner cells connected to the oocyte with GJ. GJ are intercellular connections formed by two connexons, which are assemblies of six connexins structured in a hemichannel. They allow passive diffusion of metabolites, ions, and second messengers up to a molecular mass of 1,5 kD between adjacent cells. Within the cumulus-oocyte complex (COC), GJ are present on thin membranous extensions originating from the cumulus cells connecting their cytoplasm with the oocyte. These extensions traverse the zona pellucida, a glycoprotein layer surrounding the oocyte, and are therefore called transzonal projections (TZP) (Baena and Terasaki, 2019).

In general, it is assumed that small molecules are exchanged by bidirectional communication through the GJ in bovine COCs. However, evidence of specific molecules passing through these TZP is lacking. In 2015, it was demonstrated that besides cAMP and purines (Thomas et al., 2004), nascent RNAs are crossing through these GJ (Macaulay et al., 2016). In the present study, we wanted to test the hypothesis that mature miRNAs are able to traverse through GJ in the bovine COC.

To investigate this hypothesis, we used the GJ blocker carbenoxolone (CBX) to study the miRNA content differences in oocytes with or without direct GJ contact with cumulus cells. Previous studies have demonstrated that CBX-treated oocytes induce a breakdown in gap junction communication between the oocyte and cumulus cells, resulting in a drop of cAMP levels within the oocyte and resumption of meiosis, as shown in rats (Sela-Abramovich et al., 2006), pigs (Appeltant et al., 2015), and cows (Li et al., 2016). However, CBX-blocked GJ communication negatively affects cumulus expansion, likely by reducing the concentration of oocyte-secreted factors in the cumulus cells. Furthermore, the early resumption of meiosis can negatively impact oocyte quality due to low levels of GSH, which is important during fertilization and sperm head condensation (Li et al., 2016). While the biological effects of CBX treatment on oocyte physiology are well-documented, its impact on miRNA communication within the COCs remains unexplored.

Therefore, this study focuses on the role of GJs in miRNA communication within the bovine COC, utilizing CBX to elucidate the mechanism. MiRNA sequencing was conducted to compare the miRNA content in oocytes with or without direct contact via GJ with the cumulus cells. Our objectives were to identify differentially expressed (DE) miRNAs between these groups at various stages of oocyte maturation and to visualize miRNA transfer through these GJ using a confocal microscope and carboxyfluorescein (FAM) labeled mimics. Furthermore, we aimed to validate these results with quantitative reverse transcription polymerase chain reaction (RT-qPCR) on cumulus cells originating from COCs where the oocytes were injected with the FAM-labeled mimics and cultured with or without the GJ blocker.

## 2 Results

### 2.1 Significantly differently expressed miRNAs after 1, 6 and 22 h of maturation time

Following UMI removal and de-duplication, each sample yielded between 1.9 and 13.4 million clean reads (with an average of 8.4 million). The mean quality Phred score per position consistently remained very high, exceeding 35 across all samples. A slight decline was observed towards the end of the read, typically after position 60, yet predominantly staying above 30. More information about the sequencing, including expression table and read count summary, are included in the Supplementary Material.

Differentially expressed miRNAs in the comparisons between the four groups at the three timepoints are summarized in Table 1. After 1 h of maturation, 1,066 unique miRNAs were expressed in one or more experimental groups and 802 miRNAs were expressed in all groups as shown in the Venn diagram (Supplementary Figure S1). The CBX group compared with the co-culture group had 373 reliable expressed miRNAs. Only one miRNA was significantly downregulated in the co-culture group compared to CBX: bta-novel-miR-79 (Supplementary Figure S2; Supplementary Table S1). Comparing the CBX group with the DO group, two miRNAs were DE out of 363 features: bta-novel-miR-559 and bta-miR-206, both downregulated in the DO group (Supplementary Figure S3; Supplementary Table S2). In the four other comparisons in the 1-h group (control vs. CBX, control vs. cocult, control vs. DO, and cocult vs. DO), no significantly DE miRNAs were identified.

**TABLE 1:** The differentially expressed miRNAs in all intra-group comparisons after 1, 6, and 22 h of maturation.

	1 h	6 h	22 h
Control vs. CBX			bta-novel-miR-895
			bta-novel-miR-906
			bta-miR-21-5p
			bta-novel-miR-162
			bta-novel-miR-364
Control vs. Cocult			
Control vs. DO			bta-novel-miR-906
			bta-novel-miR-321
			bta-novel-miR-318–0
			bta-novel-miR-432
			bta-novel-miR-672
			bta-novel-miR-879
CBX vs. Cocult	bta-novel-miR-79		
CBX vs. DO	bta-novel-miR-559 bta-miR-206	bta-novel-miR-824	bta-novel-miR-895
		bta-novel-miR-485	
		bta-novel-miR-318–0	
		bta-novel-miR-484	
		bta-novel-miR-457	
Cocult vs. DO		bta-novel-miR-137	
		bta-novel-miR-672	

Similar to the 1-h maturation comparison, five miRNAs were downregulated in the DO group compared with the CBX group after 6 h maturation: bta-novel-miR-824, bta-novel-miR-485, bta-novel-miR-318-0, bta-novel-miR-484, and bta-novel-miR-457 (Supplementary Figure S4; Supplementary Table S3). In total, 327 miRNAs were expressed in both DO and CBX groups. In the co-culture versus DO group, 326 miRNAs were expressed, of which two were DE: one down-regulated (bta-novel-miR-137) and one upregulated (bta-novel-miR-672) in the DO group (Supplementary Figure S5; Supplementary Table S4). Similar as in the 1-h group, four comparisons did no yield any significantly DE miRNAs after 6 h of maturation (control vs. CBX, control vs. cocult, control vs. DO, and CBX vs. cocult, Table 1). Overall, 784 miRNAs were expressed in all groups which is depicted in the Venn diagram in Supplementary Figure S6.

**FIGURE 1:**
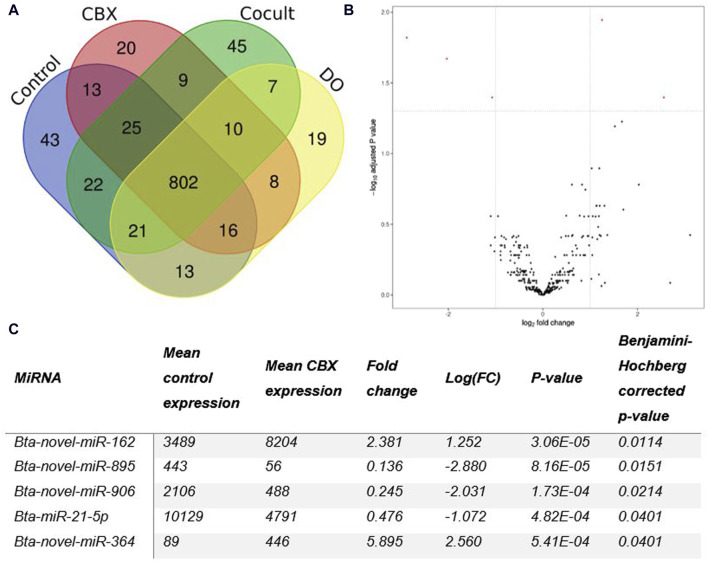
**(A)** A Venn diagram illustrating the number of miRNAs present in each group of oocytes after 22 h of maturation. **(B)** The differentially expressed miRNAs between the control group and the CBX group after 22 h of maturation are shown in a Volcano plot. Five statistically significant results (PAdj < 0.05 and |log2FC| ≥ 1) (red dots) were found: three down- and two up-regulated in the CBX group. **(C)** The table shows the details about the number of reads, fold change, and *p*-values for each of the five statistically significant miRNAs detected in the CBX group compared to the control group after 22 h of maturation. (CBX, carbenoxolone; Cocult, co-culture group; DO, denuded oocytes; FC, fold change).

Micro RNA sequencing revealed 1,073 unique miRNAs expressed in all groups after 22 h of oocyte maturation and 802 miRNAs were expressed in all different experimental groups as shown in the Venn diagram (Figure 1A). Comparing the CBX group with the control group, five DE miRNAs were found out of the 371 expressed miRNAs in both groups (Figure 1B). Three miRNAs (bta-novel-miR-895, bta-novel-miR-906 and bta-miR-21-5p) were downregulated and two (bta-novel-miR-162 and bta-novel-miR-364) were upregulated in the CBX group (Figure 1C; Supplementary Figure S7). In the comparison between control vs. DO, bta-novel-miR-906 was also downregulated in the DO group, as well as bta-novel-miR-321. Four miRNAs were upregulated in the DO group: bta-novel-miR-318-0, bta-novel-miR-432, bta-novel-miR-672, and bta-novel-miR-879 (Supplementary Figure S8; Supplementary Table S5). Bta-miR-novel-895 was downregulated in the CBX group compared with the DO group (Supplementary Figure S9; Supplementary Table S6). The other three comparisons (control vs. cocult, CBX vs. cocult, and cocult vs. DO) did not result in any significantly DE miRNAs. The genomic location and predicted seed region of the DE novel miRNAs are included in Supplementary Table S7.

Intra-group comparisons were conducted at each time point (0, 6, and 22 h) during oocyte maturation, resulting in a total of six comparisons across all groups. Notably, only one comparison revealed no DE miRNAs at all time points: control vs. co-culturing groups (Table 1). DE analysis was conducted across various time points within each group. However, the findings from this analysis do not significantly contribute to this article’s content and will be presented in another paper.

### 2.2 Pathway analysis

Candidate target genes were predicted for the significantly differentially expressed miRNAs in each inter-group comparison across various time points followed by pathway analysis based on the KEGG database and the GO terms. Supplementary Table S8 provides a comprehensive list of the KEGG terms identified in each comparison encompassing DE miRNAs with their indicative *p*-value.

In the comparison between control vs. DO group, six DE miRNAs were detected after 22 h of maturation, which resulted together in 36 overrepresented KEGG terms with the lowest *p*-value for “Human cytomegalovirus infection” and “Mannose type O-glycan synthesis.” Overrepresented pathway maps encompassed categories such as “Cancer,” “Signal Transduction,” “Immune System,” and “Endocrine System.” For the pathway analysis based on the GO terms, 145 terms were overrepresented for biological process, 22 for cellular components and 47 for molecular function. The corresponding heatmaps are included in the Supplementary Figures S10–S12. Another comparison between two control groups yielded two DE miRNAs, here after 6 h of maturation: co-culture vs. DO. Only five KEGG terms were overrepresented, with “DNA replication” and “microRNAs in cancer” as top hits. Furthermore, 82 KEGG terms were overrepresented for biological process, 21 for cellular component, and 29 for molecular function (Supplementary Figures S13–S15).

The three comparisons with CBX, all yielded DE miRNAs. For the comparison between CBX and co-culture, only one timepoint had one significant DE miRNA: 1 h of maturation. Pathway analysis on the candidate targets of this one miRNA, resulted in five overrepresented KEGG terms with “NF kappa B signaling pathway” as most prominent. GO-term analysis resulted in 40 overrepresented terms for biological process, and twelve for both cellular component and molecular function (Supplementary Figures S16–S18). Additionally, the comparison between CBX and DO yielded significant DE miRNAs in all time points. After 1 h of maturation, four KEGG terms were overrepresented, with “chemical carcinogenesis” being the top hit. After 6 h of maturation, 37 KEGG terms were overrepresented, with “pathways in cancer” having the lowest *p*-value. After 22 h of maturation, four KEGG terms were overrepresented, with “cholesterol metabolism” being the most prominent. For the GO terms for the 1-h maturation group, 84 were overrepresented for biological process, and both thirteen for molecular function and cellular component (Supplementary Figures S19–S21). Furthermore, after 6 h of maturation 97, 14, and 29 GO terms were overrepresented for biological process, cellular component, and molecular function, respectively (Supplementary Figures S22–S24). Similar for the 22-h group, 39, 7, and 15 GO terms were overrepresented for biological process, cellular component, and molecular function, respectively (Supplementary Figures S25–S27).

**FIGURE 2:**
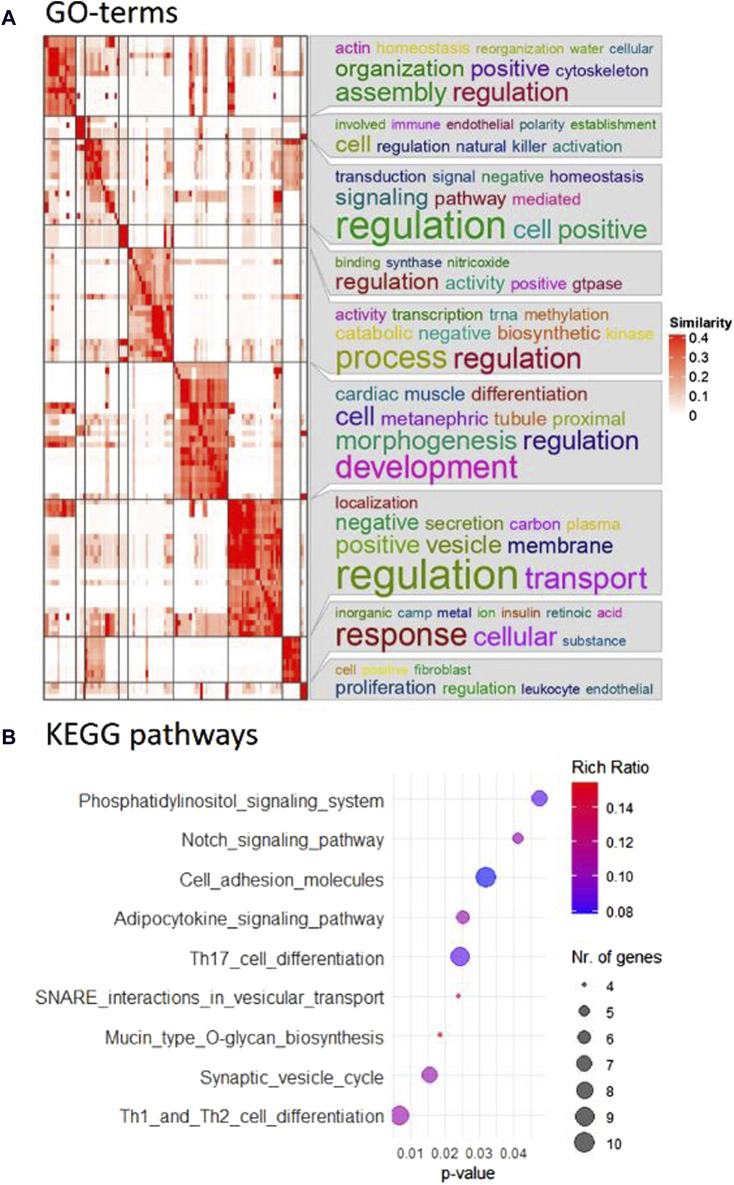
**(A)** Pathway analysis revealed 116 overrepresented biological processes. Using simplifyEnrichment in R, the corresponding GO terms were semantically clustered and represented in a heatmap. The word cloud annotation on the right side summarizes the functions of each cluster with keywords based on GO background vocabulary and font size representing significance. The color gradient from white to red corresponds to the similarity between 0–0.4. **(B)** Nine KEGG pathways were identified as overrepresented in the comparison between control and CBX groups after 22 h maturation and are included in this bubble chart. The color gradient, from blue to red, represents the rich ratio from 0.08–0.016, and the size of the bubbles represents the number of candidate genes for the differentially expressed miRNAs that were linked to these KEGG terms.

In particular, the comparison between the control group and the CBX group after 22 h of maturation was prioritized for subsequent analysis due to the specific interest in GJ functionality during oocyte maturation. “Th1 and Th2 cell differentiation,” “synaptic vesicle cycle,” and “mucin type O-glycan production” were the top hits among the nine KEGG pathways that were overrepresented (Figure 2B). One hundred and sixteen biological processes were overrepresented, according to the GO-term analysis, with “cellular water homeostasis” and “regulation of natural killer cell activation” as most prominent. All GO terms, including their indicative *p*-value are listed in Supplementary Table S9. The corresponding heatmap is shown in Figure 2A. The GO terms for cellular component and molecular function are listed in Supplementary Tables S10, S11, with their corresponding heatmaps in Supplementary Figures S28, S29, respectively. “GPI anchor transamidase complex” was the first GO term out of eleven significant GO terms for cellular component, and “water channel activity” had the lowest *p*-value from the 37 significantly overrepresented GO terms for molecular function.

### 2.3 Labeled bta-miR-21-5p mimics cross through the gap junctions

Among the five DE miRNAs identified in the comparison between the control and CBX groups after 22 h of maturation, only one, bta-miR-21-5p, had been previously described. Consequently, this particular miRNA was chosen as a proof-of-concept for the GJ functionality experiments.

**FIGURE 3:**
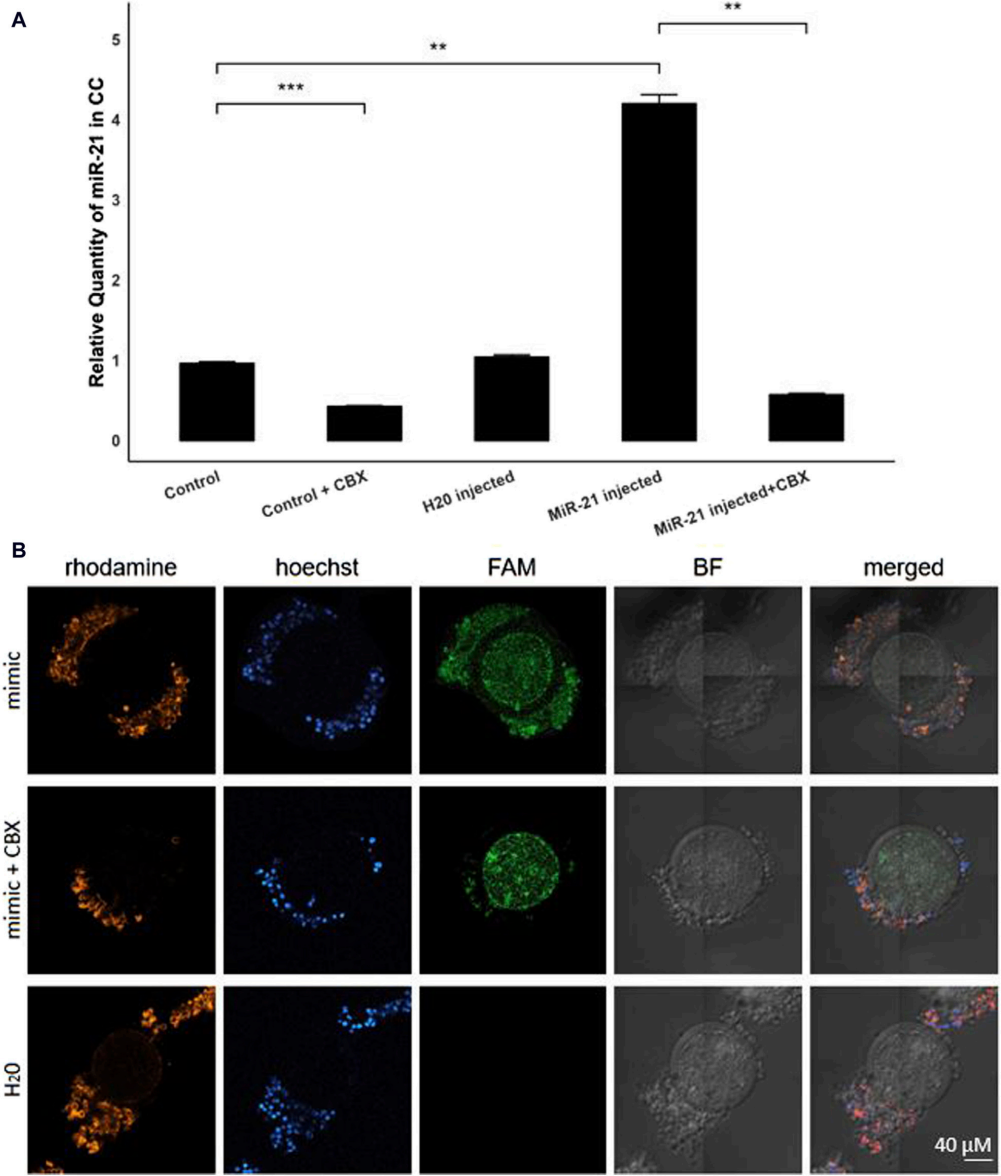
Bta-miR-21-5p expression after micro-injection with bta-mir-21-5p mimics. **(A)** The relative expression levels of bta-miR-21-5p between the cumulus cells from the control group and the cumulus cells from the bta-mir-21-5p injected group, both with or without CBX in the maturation medium and the H20 injected group. Data are represented as mean ± SEM of triplicates from three experiments. **(B)** Bright-field and fluorescence images show the FAM fluorescence from the mimic present in the injected oocytes. The cumulus cells from COCs matured in the presence with CBX have less FAM fluorescence. Hoechst and rhodamine staining visualized the nucleus and GJ network, respectively. All pictures are shown at the same magnification, indicated in the bottom right corner. (CC, cumulus cells; CBX, carbenoxolone; mimic, FAM labeled miR-21 mimic; FAM, carboxyfluorescein; BF, bright field).

By injecting FAM-labeled bta-miR-21-5p mimics into the oocytes before entering the maturation process, with or without the presence of CBX, the intracellular trafficking of microRNAs through GJ could be elucidated. RT-qPCR analysis unveiled a four-fold elevation (*p* = 0.001) in the levels of bta-miR-21-5p within the cumulus cells of COCs that received the bta-miR-21-5p mimic injection as compared to the cumulus cells from non-injected COCs, as depicted in Figure 3A. This observation serves as compelling evidence of the migration of miRNA mimics from the oocyte to the cumulus cells. Importantly, the heightened representation of bta-miR-21-5p within the cumulus cells of the bta-miR-21-5p-injected COCs was diminished when these complexes were subjected to maturation in the presence of CBX (*p* = 0.0012), demonstrating the prevention of miRNA mimic transport through GJs. Furthermore, the expression of bta-miR-21-5p in the cumulus cells of non-injected oocytes was found to be reduced by half (*p* = 0.0006) when these oocytes were cultured in the presence of CBX, as opposed to those cultured under standard conditions. Utilizing confocal microscopy in conjunction with Phalloidin Rhodamine TRITC staining, it became evident that the miRNA mimics were distributed within the oocyte, cumulus cells, and the GJ, thereby confirming their intracellular localization (Figure 3B).

## 3 Discussion

MiRNAs have emerged as critical regulators during oocyte maturation, exerting their influence by modulating gene expression in a temporal- and spatial-specific manner. These small non-coding RNAs play a pivotal role in various processes essential for oocyte development (Bartel, 2009). In this study, we aimed to investigate the miRNA profile during bovine oocyte maturation and ascertain whether miRNA transfer occurs through GJ.

The GJ network plays a critical role in the functional coordination of cells within the COC. GJ facilitate the direct exchange of small molecules, including ions, metabolites, and second messengers, between adjacent cells (Lemcke et al., 2015). Earlier, the transfer of miRNAs through GJ was demonstrated in HeLa cell lines by transfecting with FAM-labeled mouse miRNAs. Notably, no discernible variance in permeability was observed among various miRNAs. Subsequent investigations revealed that this communication is connexin-dependent, particularly involving Cx43. This is the primary GJ protein expressed in bovine COCs which exhibits the highest permeability for miRNAs (Zong et al., 2015) and displays a predilection for negatively charged molecules (Elfgang et al., 1995).

The transfer of miRNAs between cells within the COC has been postulated as a crucial mechanism for intercellular communication (Lemcke et al., 2015). This bidirectional signaling in the ovarian follicle can occur either directly, using a network of GJ (juxtacrine) as we demonstrated here or from a distance (autocrine, endocrine, and paracrine). Paracrine signaling occurs in the follicle by releasing miRNA-loaded extracellular vesicles or Argonaute 2 bounded miRNAs into the follicular fluid (Sohel et al., 2013). In the present paper, the specific focus was on exploring the juxtacrine form of communication by selectively blocking GJ. This approach ensures that any differentially expressed miRNA observed was likely traversing through these channels, as other transport methods would still be operational in all experimental groups.

Through the inhibition of GJ functionality with CBX and various control groups, we probed the bovine miRNA profile at different time points of *in vitro* oocyte maturation. Our results reveal significant differences in the expression of miRNAs between several groups at all time points (1, 6, and 22 h) of maturation, with eighteen identified DE miRNAs across all comparisons. Intriguingly, sixteen of these were novel predicted miRNAs, underscoring the existence of numerous undiscovered bovine miRNAs. Four novel miRNAs were significantly differently expressed in two comparisons: bta-novel-miR-906, bta-novel-miR-895, bta-novel-miR-318-0, and bta-novel-miR-672. Firstly, bta-novel-miR-906 was downregulated in both the CBX and DO group compared to the control after 22 h of maturation, suggesting that this miRNA is less expressed without the presence of (gap junctional contact) from cumulus cells. Additionally, bta-novel-miR-895 exhibited downregulation in the CBX group compared to both the control group and the DO group after 22 h of maturation. This outcome indicates that the reduced expression of this miRNA is unlikely attributed to the absence of GJ communication with cumulus cells, but rather indicates a regulatory effect of closed GJs that persists when compared to denuded oocytes. The third miRNA differentially expressed in two comparisons is bta-novel-miR-318-0, which is downregulated in the DO group compared to the CBX group after 6 h of maturation and downregulated in the control group compared to the denuded group after 22 h of maturation. This dual downregulation pattern suggests a complex regulatory mechanism involving gap junction functionality and maturation time, wherein the presence of open gap junctions in control oocytes contributes to a distinct miRNA expression profile compared to denuded oocytes. The fourth miRNA is bta-novel-miR-672, which exhibited downregulation in the DO group compared to both the co-culture group and the control group after six and 22 h of maturation, respectively. This implies that the expression of this miRNA is reduced in the absence of cumulus cells, whether in direct contact or nearby.

As expected, there was an increased detection of DE miRNAs at later time points, which may be attributed to the extended duration, allowing the CBX or culturing method more time to exert its influence. Given our particular interest in GJ functionality during oocyte maturation, the five miRNAs demonstrating up- or downregulation between oocytes with open GJs and those with blocked GJs after 22 h of maturation became our primary focus.

Particularly noteworthy is the twofold upregulation of bta-miR-21-5p in the control group compared to the CBX experimental groups after 22 h of maturation. This observation indicates the potential role of bta-miR-21-5p in the bidirectional communication between oocytes and surrounding cumulus cells. Previous studies in human COCs have implicated bta-miR-21-5p as a key regulator of cumulus cell viability and oocyte maturation, likely through its interaction with the *PTEN* gene (Bartolucci et al., 2020). This gene suppresses the AKT/phosphatidylinositol 3-kinase (PI3K) -dependent survival pathway in the cumulus cells, inhibiting apoptosis (Han et al., 2017). In mice, this pathway was demonstrated to have a stage-specific control function of the follicular activation (Jagarlamudi et al., 2009). Our findings further support the functional significance of bta-miR-21-5p in these processes.

The four other miRNAs that exhibited differential expression between oocytes with open and blocked GJ after 22 h of maturation were bta-novel-miR-895, bta-novel-miR-906, bta-novel-miR-162, and bta-novel-miR-364. Firstly, bta-novel-miR-895 was downregulated in the CBX group and lacks an identical seed region in other species. It resides on the ninth chromosome in the bovine genome (Supplementary Table S7). Bta-novel-miR-906, also downregulated in the CBX group, is positioned on the mitochondrial chromosome. Notably, an identical seed region was found in equine: eca-miR-9117, previously identified in testis samples (Platt et al., 2014). The third identified novel miRNA is bta-novel-miR-162, which displayed upregulation in the CBX group. This miRNA’s predicted seed region is similar to mmu-miR-8092, only once identified in mouse spleen (Polikepahad and Corry, 2013). In the bovine genome, it is situated on the 13th chromosome. Lastly, bta-novel-miR-364, located on the 19th chromosome, shares an identical seed with hsa-miR-4634 in human. This miRNA, which was upregulated in the CBX group, has been described as a biomarker for detecting lymph node metastasis in stage III colorectal carcinoma (Xu et al., 2014), as well as an early-stage breast cancer biomarker in serum (Shimomura et al., 2016).

Moreover, pathway analysis across different time points for all comparisons encompassing the CBX group revealed several overrepresented KEGG pathways, shedding light on the dynamic changes in cellular signaling and metabolism in response to CBX treatment. For instance, at 1 h of maturation, KEGG terms such as “Pathways in cancer” and “Chemical carcinogenesis” were prominently overrepresented in the CBX vs. co-culture group, “Glioma” in CBX vs. DO group, and “MicroRNAs in cancer” in both previous comparisons. This indicates early cellular stress responses to this CBX treatment. At 6 h, pathways like “Thyroid cancer” and “Melanoma” in CBX vs. DO group highlighted continued stress and immune-related responses. By 22 h, pathways involved in “GnRH secretion,” “Sulfur metabolism,” and “SNARE interactions in vesicular transport” were notably overrepresented, suggesting a shift towards metabolic and vesicular transport processes. A possible explanation for this finding, is the natural time for gap junction closure, found to be close to the LH surge during ovulation (around 6–8 h of *in vitro* maturation) (Gilula et al., 1978; Norris et al., 2010).

Two comparisons between control groups also revealed significant DE miRNAs: co-culture vs. DO group after 6 h of maturation and control vs. DO group after 22 h of maturation. Pathway analysis of their predicted targets identified 41 overrepresented KEGG terms. The prominence of immune system and cancer-related pathways, such as “Intestinal immune network for IgA production” and “melanoma” in the control vs. DO group, and “MicroRNAs in cancer” in co-culture vs. DO group, suggests that the absence of cumulus cells may trigger stress responses similar to those observed in immune dysregulation and carcinogenesis. These stress responses are expected since the developmental potential of oocytes denuded before maturation is significantly lower compared to control COCs. However, this developmental potential is largely restored when co-cultured with intact COCs (Luciano et al., 2005). The overrepresentation of “Mannose type O-glycan biosynthesis” pathway suggests alterations in protein glycosylation, which could affect cell adhesion and function. This was demonstrated in mice lacking N- and O-glycans, where the cumulus expansion was modified with lower levels of hyaluronan and inter-α-inhibitor heavy chains (Lo et al., 2019). Additionally, the impact on various signal transduction pathways, such as “Estrogen signaling pathway,” “Prolactin signaling pathway,” and “Neurotrophin signaling pathway” underscores the potential disruption of critical cellular signaling networks due to the absence of (direct contact of) cumulus cells.

The results of the pathway analysis between the control group and the CBX group after 22 h of maturation provided valuable insights into the underlying biological processes that are differentially regulated. Notably, among the nine KEGG pathways that were significantly overrepresented, “Th1 and Th2 cell differentiation” emerged as a top hit, highlighting the importance of these cells during oogenesis and probably early pregnancy. Th2 cells become predominant, secreting cytokines like IL-4 and IL-10 to establish an anti-inflammatory environment. This shift suppresses Th1-driven immune responses, creating a favorable milieu for fertilization and embryo elongation (Reinhard et al., 1998). Further, the predominance of “mucin type O-glycan production” is noteworthy, which suggests potential alterations in glycosylation processes. This confirms earlier reports that inhibition of protein glycosylation can increase trafficking of Cx43 to the plasma membrane, its phosphorylation and its opening indirectly via a cAMP pathway (Johnstone et al., 2012).

By labeling a bta-miR-21-5p mimic with FAM, we visualized the transfer of this specific miRNA through GJ within the COC and further validated it with RT-qPCR. The four-fold increase in bta-miR-21-5p levels within cumulus cells following mimic injection provides compelling evidence of miRNA migration from oocytes to cumulus cells through the GJ. This observation confirms that miRNAs can traverse the GJ network, establishing a direct molecular pathway for communication between oocytes and cumulus cells. The transfer of miRNAs through GJ has been previously reported in other cell types and biological contexts (Zong et al., 2015; Peng et al., 2019), further supporting the concept of miRNA-mediated intercellular communication through GJ.

While our primary objective was to investigate the miRNA profile and communication within the COC through GJ, an interesting finding emerged regarding the impact of co-culturing. We compared the miRNA profiles of oocytes from COCs cultured independently (control group) with those from DOs co-cultured with COCs but without direct contact. Surprisingly, no significantly differently expressed miRNAs were observed between these groups at all different maturation times. This result implies that the co-culturing approach might mitigate the negative impact associated with the removal of cumulus cells, even without direct contact. Furthermore, this suggests that the lack of juxtacrine communication may be compensated for by alternative forms of long-distance signaling, such as paracrine signaling within the maturation medium. This aligns with previous studies indicating that denuded oocytes before maturation have a limited developmental potential, and the presence of solely CCs does not improve their developmental competence. However, co-culturing with intact COCs partially restores the developmental capability of the DOs (Luciano et al., 2005).

This co-culturing strategy aligns with previous studies that have emphasized the importance of paracrine signaling and intercellular communication between oocytes and cumulus cells (Luciano et al., 2005; Sun et al., 2015). Even when direct physical contact between oocytes and cumulus cells was absent, the presence of COCs within the culture environment seemed to provide a supportive milieu that partially compensated for the absence of cumulus cells. Earlier findings underscored the relevance of this compensatory effect, particularly during fertilization, when the demand for effective cumulus cell communication is most pronounced (Ortiz-Escribano et al., 2016).

Hence, future experiments involving supplementation of isolated extracellular vesicles (EVs) during maturation could yield further insights and elucidate the respective roles of gap junctions and EVs in oocyte maturation. Another intriguing avenue for future research could be the incorporation of cumulus cells into RNA sequencing analysis, potentially with a focus on distinguishing between inner and outer cumulus cells. This approach has the potential to expand our understanding and refine our knowledge in this area.

In conclusion, our study provides compelling evidence that communication with miRNAs within the bovine cumulus-oocyte complex is facilitated by the gap junctional network. The differential expression of miRNAs, including bta-miR-21-5p, and the visualization of bta-miR-21-5p transfer through GJ shed light on the complex interplay between oocytes and cumulus cells during oocyte maturation. Further investigations into the molecular mechanisms of miRNA-mediated communication are warranted to elucidate their functional significance and potential therapeutic applications in the field of reproductive biology.

## 4 Materials and methods

### 4.1 Design experiment 1

**FIGURE 4:**
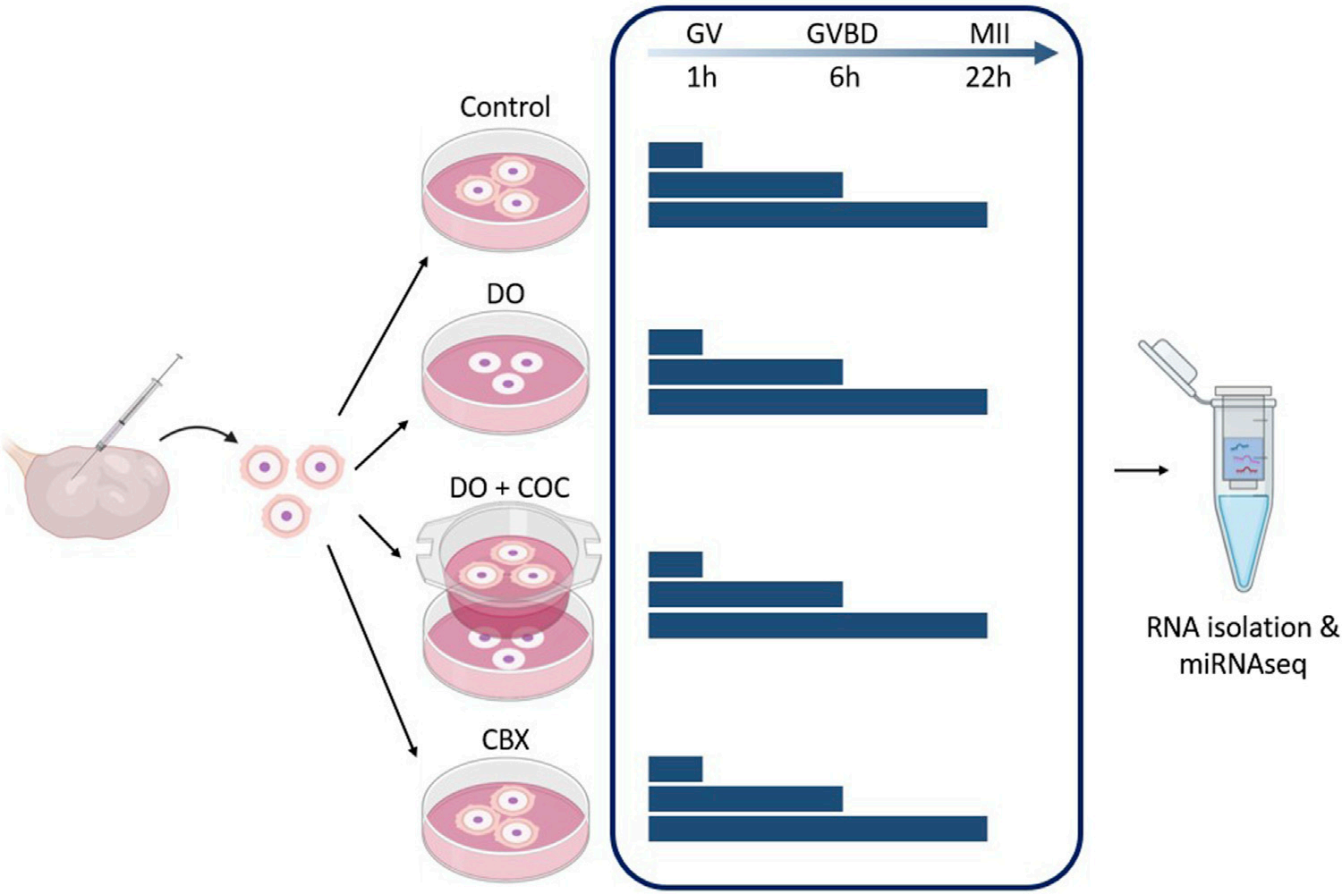
Experimental design 1: influence of gap junctions (GJ) on miRNA profile of oocytes and cumulus cells. Carbenoxolone (CBX), a GJ blocker, was added to the maturation medium of one group. As controls, there was a normal condition group (Control), a group with denuded oocytes (DO) and a group of denuded oocytes with COCs in co-culture (DO + COC). Three replicates from each group were matured for 1, 6, and 22 h before total RNA isolation and miRNA sequencing. (GV, germinal vesicle; GVBD, germinal vesicle breakdown; MII, metaphase II stage). Created with Biorender.com.

In Figure 4, a schematic overview of the different experimental groups is given. There were four experimental groups: COC, denuded oocytes (DO), coculturing of DOs and COCs (DO + COC), and carbenoxolone (CBX; 3β-hydroxy-11-oxoolean-12-en-30-oic acid 3-hemisuccinate) treated group. Each of these experimental setups had maturation periods of 1, 6, and 22 h, to receive oocytes at germinal vesicle, germinal vesicle breakdown and metaphase II stage (Motlík et al., 1978). Subsequently, RNA samples were isolated for miRNA sequencing, and in each experimental condition, one group underwent *in vitro* fertilization and subsequent culturing to serve as a control. For each maturation time point, each experimental group consisted out of 15 oocytes (co-cultures had an extra 15), and an extra 15 for fertilization. This experiment was repeated six times. Before RNA isolation, two replicates consisting out of 15 oocytes were pooled to obtain 30 oocytes in each group.

#### 4.1.1 Sample preparation and *in vitro* maturation

Tissue culture media (TCM)-199-medium, gentamycin, and kanamycin were obtained from Life Technologies Europe (Ghent, Belgium). All other chemicals not otherwise listed were purchased from Sigma-Aldrich (Diegem, Belgium). All media were filtered before use (0.22 µm filter, Pall Corporation, Ann Arbor, MI, United States).

Bovine ovaries were obtained from a local slaughterhouse and transported in an insulated box within 1 h after collection. Upon arrival, ovaries were rinsed in physiological saline supplemented with kanamycin (5 mg/mL) and wiped with absolute ethanol. COCs were aspirated from ovarian follicles with a diameter of 2–8 mm with an 18 G needle. Only multilayered COCs containing dark cytoplasm were selected and washed with Hepes buffered Tyrode’s albumin lactate pyruvate (Hepes-TALP). Next, half of the collected COCs were vortexed for 8 min in Hepes-TALP supplemented with 0.1% hyaluronidase to obtain DOs. COCs and DOs were matured in groups of 15 in 500 µL serum-free TCM 199 medium supplemented with gentamicin (50 mg/L), epidermal growth factor (20 μL/mL), with or without 100 µM of CBX (Merck, Darmstadt, Germany). Preliminary studies using Lucifer Yellow injections were performed to determine the most efficient concentration of CBX (50 or 100 µM), which is a glycyrrhetinic acid derivative known to block GJ (Rozental et al., 2001). Coculturing of DOs with COCs (15:15) was done on a distance, so-called non-contact co-culture, using a ThinCert™ with pores of 3 µm (Greiner Bio-one, Vilvoorde, Belgium).

#### 4.1.2 Cell isolation

After maturation, the COCs were vortexed for 5 min in 0.1% hyaluronidase to separate the oocytes and cumulus cells, and DOs were treated with pronase 0.1% (w/v) for 20 s to remove the zona. Oocytes containing a visual polar body were selected and washed in Hepes-TALP before being preserved in lysis buffer at −80°C until further extractions. By aggregating two replicates, the cumulative count of oocytes in each of the three replicates amounted to a total of 30 oocytes.

#### 4.1.3 Total RNA extraction

Total RNA was extracted from oocytes using the miRNeasy Mini kit (Qiagen, Germantown, United States). Briefly, cells were disrupted with QIAzol Lysis Reagent and deproteinized with chloroform. Subsequently, the upper aqueous phase was collected and mixed with 100% ethanol. After centrifugation steps in the RNeasy mini spin column, the RNA was eluted in RNase-free water. To ensure the samples are DNA free, they were treated with RNase-Free DNase Set (Qiagen, Germantown, United States).

#### 4.1.4 *In vitro* fertilization

The concentration of bovine frozen-thawed spermatozoa, isolated with a 45%–90% Percoll gradient was calculated using a Bürker counting chamber. These spermatozoa were diluted to a final concentration of 106 spermatozoa/mL to fertilize a control group of non-denuded COCs. This fertilization occurred in the IVF-TALP medium supplemented with bovine serum albumin (BSA) (6 mg/mL) and heparin (25 mg/mL) for 21 h.

#### 4.1.5 *In vitro* culture

All zygotes were vortexed for 3 min to remove the redundant spermatozoa and cumulus cells, followed by washing steps with TALP buffered Hepes. Presumptive zygotes were cultured in groups of fifteen in 50 µL synthetic oviductal fluid, which was supplemented with insulin (5 μg/mL), transferrin (5 μg/mL), selenium (4 mg/mL), and bovine serum albumin (4 mg/mL), covered with paraffin oil (SAGE, Cooper Surgical, Trumbull, CT, United States). This incubation was performed for 8 days in conditions with 5% CO2, 5% O2, and 90% N2, and with a temperature of 38.5°C. Cleavage and blastocyst rates were evaluated 2 and 8 days post insemination. These blastocysts only served as a control for normal conditions and were therefore discarded after 8 days of culturing without any further proceedings.

#### 4.1.6 RNA sequencing and data analysis

For each sample, a sequencing library was constructed using the QIAseq miRNA Library Kit (Qiagen, Germantown, United States), which included a nineteen nucleotides fixed sequence spacer, and a unique twelve nucleotides molecular identifier at the 3′-end of each forward read. Subsequently, paired-end 150 sequencingwas performed on a NovaSeq device (Illumina) by service provider Genewiz (Azenta Life Sciences). Although paired-end 150 is certainly not required for miRNA sequencing, this was the cheapest option offered by the service provider. All downstream procedures were done using the forward read only.

Subsequent to adaptor trimming with UMI-tools (v1.1.1) (Smith et al., 2017), the sequences were subjected to quality and contamination evaluations using FastQC (0.11.9) (Andrews, 2010) and fastQ Screen (v0.15.1) (Wingett and Andrews, 2018), respectively. The mapped alignment was performed against the ARS-UCD1.2 bovine genome (ENSEMBL release 105). Identification of known bovine miRNA and the prediction of novel miRNAs was performed with the miRPro pipeline (v.1.1.4) (Shi et al., 2015) by annotating the aligned and deduplicated sequence reads against the ARS-UCD1.2 bovine genome (ENSEMBL release 105) and against the matured miRNAs from the miRbase database (release 22.1).

Differential expression analysis was done in R (v4.1.2) using the edgeR (v3.36) package (Robinson et al., 2010). TMM-normalized feature counts were fitted with the quasi likelihood model and comparisons at the gene level were done with the F-test. The ovary pool was included as a batch effect and *p*-values were corrected for repeated testing using the Benjamini–Hochberg method. Results were marked as significant when the fold change of two consecutive time points yielded a q-value (corrected *p*-value) < 0.05 and |log2FC| ≥ 1.

#### 4.1.7 Small RNA annotation and pathway analysis

To predict the candidate target genes for the significantly DE miRNAs, miRanda (v.3.3a) was used on miRNA and isolated 3′UTR sequences. In addition, the functional analysis was conducted on the refined set of target genes after filtering using the following thresholds: minimum score = 155 and maximum energy = −20. This analysis was done in R (v4.1.2) using the fgsea (v1.20.0), EnrichmentBrowser (v2.24.2), and simplifyEnrichment (v1.4.0) packages. The Benjamini–Hochberg corrected *p*-values < 0.05 were considered statistically significant.

### 4.2 Design experiment 2

**FIGURE 5:**
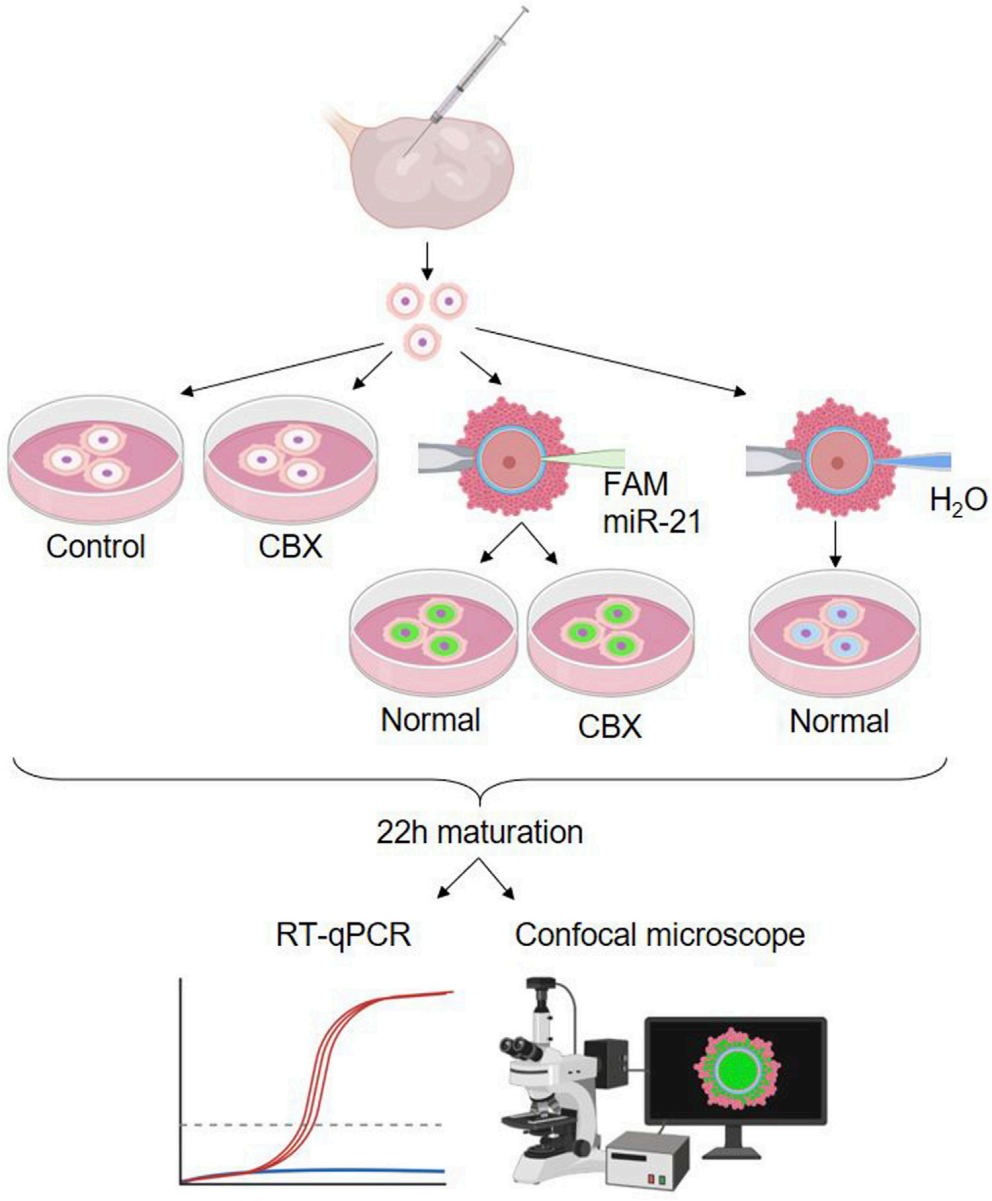
Experimental design 2: passage of miR-21 through gap junctions. Showing the five different experimental groups: a non-injected control group, non-injected group cultured in carbenoxolone (CBX), two carboxyfluorescein (FAM) labeled bta-miR-21-5p mimic injected groups cultured with or without CBX and one group injected with H20, the solvent. (RT-qPCR: quantitative reverse transcription polymerase chain reaction). Created with Biorender.com.

The second experimental design included five different experimental groups: two non-injected control groups, cultured with or without CBX, two FAM labeled bta-miR-21-5p mimic injected groups cultured with or without CBX, and one group injected with H20, the solvent. A schematic overview of these groups is shown in Figure 5. A single replicate consisting of 15 oocytes for each group was used for fluorescent staining, while three replicates, each consisting of 15 oocytes, were used for RT-qPCR.

#### 4.2.1 Microinjection of miRNA mimics

Bta-miR-21-5p mimics labeled with carboxyfluorescein (FAM) (Qiagen, Germantown, United States) were dissolved in sterile RNase-free water with a final concentration of 25 µM and stored as aliquots in −20°C. The injections were performed as described by (Tan et al., 2020) using an inverted bright field Nikon TE300 microscope (Nikon Benelux). In short, partially denuded immature (germinal vesicle stage) oocytes were transferred to a 5 µL drop of Hepes buffered medium overlaid by paraffin oil in a 60 mm dish. The microinjection needle with an inner diameter of 4.3–4.9 µm (CooperSurgical) was loaded with 5 µL miRNA mimics or sterile RNase-free (control) and installed in a 30° angle. Using a Femtojet injection system (Eppendorf) the partially denuded oocytes were injected for 0.2 s at a pressure of 120 hpa. Non-injected COCs were used as a control group, and each group contained 15 COCs per replicate (n = 3).

#### 4.2.2 Cell isolation and total RNA extraction

Injected COCs, and non-injected controls were matured for 22 h as described above. One replicate was fixed for staining and visualization. For the other triplicates, the cumulus cells were collected by manually denuding the oocytes in droplets of 0.1% hyaluronidase, and stored in lysis buffer at −80°C for quantitative analysis.

#### 4.2.3 Reverse transcription real-time quantitative PCR (RT-qPCR)

Total RNA was reverse transcribed with the miRCURY LNA RT kit (Qiagen, Germantown, United States). The total reaction volume of 9.5 µL consisted of 2 µL ×5 miRCURY RT Reaction buffer, 2.5 µL RNase-free water, 1 µL ×10 miRCURY RT Enzyme Mix and 4 µL RNA (5 ng/μL). This reaction included an incubation step for 60 min at 42°C, followed by an inactivation step for 5 min at 95°C. Subsequently, RT-qPCR was performed with BioRad CFX96 PCR in a volume of 10 µL using the miRCURY LNA SYBR green PCR kit RT (Qiagen, Germantown, United States). This volume consisted of 5 µL ×2 miRCURY SYBR Green mastermix, 1 µL PCR primer mix, 1 µL RNase-free water and 3 µL diluted cDNA. After a 2-min heat activation step at 95°C, 40 cycles were executed, including 10 s denaturation at 95°C and 60 s combined annealing and extension at 56°C. Melting curve analysis was performed between 60°C–95°C. For normalization, miR-92a and miR-93 were found most stable and selected as references after testing miR-127, miR-92a, miR-93, let-7, and U6 on cumulus samples. To analyze the data, geNorm normalization was applied in qbase+. All RT-qPCR reactions were performed in triplicate, and the used primers are listed in Supplementary Table S12.

#### 4.2.4 Fixation and fluorescent staining

Oocytes were washed in Hepes-TALP and fixed overnight in 4% paraformaldehyde. Then, they were permeabilized for 10 min in 0.5% Triton X-100% and 0.05% Tween 20 in DPBS and blocked for 30 min in 1% BSA in DPBS. Further, oocytes were incubated in a dilution 1:40 Phalloidin Rhodamine TRITC (Hellobio, Ireland) for 30 min and incubated in a dilution 1:1,000 Hoechst 33342 in PBS-BSA for 10 min, both at RT in the dark. Images were captured using a Zeiss LSM9000 confocal microscope (Zaventem, Belgium) equipped with a ×40 oil immersion objective lens. The GJ network was visualized using the red channel for rhodamine-TRITC, nuclei were stained with Hoechst and visualized using the blue channel, and miRNAs were detected using the green channel for FAM labels. Additionally, bright field (BF) images were obtained to provide an overall view of the sample morphology. Unless stated otherwise, all staining products were purchased from Merck, Darmstadt, Germany.

#### 4.2.5 Statistical analysis

Data were collected from three independent experiments and are presented as mean ± standard deviation. We performed data analysis using ANOVA, followed by a two-tailed Student’s *t*-test in Excel. Results with a *p*-value of less than 0.05 were considered statistically significant.

## Data Availability

The data presented in the study are deposited in the NCBI GEO repository, accesion number GSE252748.
