# Supplementary material for: Expression profile and gap-junctional transfer of microRNAs in the bovine cumulus-oocyte complex
Source: Front Cell Dev Biol. 2024 Jul 11;12:1404675. doi: 10.3389/fcell.2024.1404675 (PMC11269113; doi:10.3389/fcell.2024.1404675)
Supplement: Supplementary file 3 [file DataSheet1.docx]

Supplementary Material

# Supplementary Tables

**Table S1:** The differentially expressed miRNA between the CBX group and the co-culturing group after one hour of maturation

| **Gene id** | **Mean CBX**  **expression** | **Mean co-cult expression** | **Fold Change** | **Log(FC)** | **P-value** | **Benjamini-Hochberg corrected p-value** |
| --- | --- | --- | --- | --- | --- | --- |
| Bta-novel-miR-79 | 2583 | 758 | 0.251 | -1.994 | 9.27E-06 | 0.00346 |

**Table S2**: The differentially expressed miRNAs between the CBX group and the DO group after one hour of maturation

| **Gene id** | **Mean CBX**  **expression** | **Mean DO expression** | **Fold Change** | **Log(FC)** | **P-value** | **Benjamini-Hochberg corrected p-value** |
| --- | --- | --- | --- | --- | --- | --- |
| Bta-novel-miR-559 | 6669 | 2282 | 0.314 | -1.671 | 1.667E-05 | 0.00605 |
| Bta-miR-206 | 451 | 80 | 0.224 | -2.157 | 0.000244 | 0.0443 |

Table S3: The differentially expressed miRNAs between the CBX group and the DO group after six hours of maturation

| **Gene id** | **Mean CBX**  **expression** | **Mean DO expression** | **Fold Change** | **Log(FC)** | **P-value** | **Benjamini-Hochberg corrected p-value** |
| --- | --- | --- | --- | --- | --- | --- |
| Bta-novel-miR-824 | 1533 | 554 | 0.387 | -1.369 | 4.182E-06 | 0.00137 |
| Bta-novel-miR-485 | 3692 | 1422 | 0.436 | -1.196 | 1.109E-05 | 0.00181 |
| Bta-novel-miR-318-0 | 1541 | 803 | 0.475 | -1.074 | 0.000111 | 0.0121 |
| Bta-novel-miR-484 | 761 | 263 | 0.389 | -1.363 | 0.000166 | 0.0136 |
| Bta-novel-miR-457 | 927 | 415 | 0.452 | -1.147 | 0.000343 | 0.0224 |

Table S4: The differentially expressed miRNAs between the co-culture group and the DO group after six hours of maturation

| **Gene id** | **Mean Co-cult**  **expression** | **Mean DO expression** | **Fold Change** | **Log(FC)** | **P-value** | **Benjamini-Hochberg corrected p-value** |
| --- | --- | --- | --- | --- | --- | --- |
| Bta-novel-miR-672 | 121 | 449 | 3.809 | 1.930 | 3.115E-06 | 0.00102 |
| Bta-novel-miR-137 | 396 | 149 | 0.382 | -1.389 | 0.000224 | 0.0365 |

Table S5: The differentially expressed miRNAs between the control group and the DO group after 22 hours of maturation

| Gene id | Mean control  Expression | Mean DO expression | Fold change | Log(FC) | P-value | Benjamini-hochberg corrected p-value |
| --- | --- | --- | --- | --- | --- | --- |
| Bta-novel-mir-906 | 2126 | 756 | 0.363 | -1.461 | 1.625e-07 | 5.573e-05 |
| Bta-novel-mir-318-0 | 841 | 1951 | 2.238 | 1.162 | 3.262e-05 | 0.00373 |
| Bta-novel-mir-321 | 2344 | 1062 | 0.488 | -1.034 | 6.652e-05 | 0.00570 |
| Bta-novel-mir-432 | 205 | 723 | 2.783 | 1.477 | 0.00101 | 0.0385 |
| Bta-novel-mir-672 | 149 | 458 | 2.895 | 1.533 | 0.000838 | 0.0385 |
| Bta-novel-mir-879 | 198 | 488 | 2.640 | 1.401 | 0.000922 | 0.0385 |

Table S6: The differentially expressed miRNA between the CBX group and the DO group after 22 hours of maturation

| **Gene id** | **Mean CBX**  **expression** | **Mean DO expression** | **Fold Change** | **Log(FC)** | **P-value** | **Benjamini-Hochberg corrected p-value** |
| --- | --- | --- | --- | --- | --- | --- |
| Bta-novel-miR-895 | 56 | 754 | 11.855 | 3.567 | 4.204E-05 | 0.0140 |

**Table S7**: Genomic locations for differentially expressed novel miRNAs

| **miRNA name** | **Predicted mature : seed** | **Predicted mature : sequence** | **Genomic location from predicted seed in bovine genome** |
| --- | --- | --- | --- |
| bta-novel-miR-79 | mml-miR-31-5p | UGCAAGAUUUUAAGCAUGACCUU | Chr11: 97182562-97182675 (-) |
| bta-novel-miR-137 | mml-miR-192-3p | UGCCAAUUCAGGGGACACGGGUUUG | Chr13: 33386482-33386596 (-) |
| bta-novel-miR-162 | mmu-miR-8092 | UCCAUCUGGACGGCUUGGCAUUGGG | Chr13: 76526680-76526794 (-) |
| bta-novel-miR-318-0 | hsa-miR-1292-3p | GCGCGCCCCCGCCGCGCG | Chr17: 69639696-69639803 (+) |
| bta-novel-miR-321 | mmu-miR-12184-3p | UCAGACGGUAAAGCAUCUGCCUG | Chr17: 26339280-26339394 (-) |
| bta-novel-miR-364 | hsa-miR-4634 | GGGCGCGAAGCGGGGCUG | Chr19: 56554303-56554410 (-) |
| bta-novel-miR-432 | chi-miR-3432-5p | UGCGGGAUCUUUAGUUGUGG | Chr21: 21809645-21809752 (+) |
| bta-novel-miR-457 | hsa-miR-4707-5p | UCCCCGGCACCUCCACCA | Chr22: 58542346-58542453 (+) |
| bta-novel-miR-484 | hsa-miR-6747-5p | GGGGGUGUAGCUCAGUGG | Chr23: 30149569-30149678 (+) |
| bta-novel-miR-485 | ppy-miR-613 | GGGGAUGUAGCUCAGUGG | Chr23: 30170729-30170841 (+) |
| bta-novel-miR-559 | hsa-miR-4783-5p | GGCGCGCCCCCGCCGCGC | Chr27: 6219131-6219238 (-) |
| bta-novel-miR-589 |  | UUGAUUUGCAUUUCUCUGA | Chr29: 5614595-5614709 (-) |
| bta-novel-miR-672 |  | GACCGAAGUGGGGAAAGG | Chr3: 2472195-2472302 (+) |
| bta-novel-miR-723 | cfa-miR-8813 | UACAGGCUUGUUAGGUCA | Chr4: 97883770-97883877 (+) |
| bta-novel-miR-824 | eca-miR-8919 | GUCAGGAUGGCCGAGUGGUCUAAGG | Chr7: 42660374-42660486 (+) |
| bta-novel-miR-879 | hsa-miR-3692-5p | UCUGCUGGAACUGCAACU | Chr8: 63779353-63779460 (-) |
| bta-novel-miR-895 |  | GCGCCCCCGGGGCCGCGG | Chr9: 93405029-93405136 (+) |
| bta-novel-miR-906 | eca-miR-9117 | GAGGGAUCCCUGCCUAGCGGG | ChrM: 16169-16279 (-) |

Table S8: KEGG pathway analysis listed from all comparisons

| Group | Pathway | p-value | Comparison |
| --- | --- | --- | --- |
| **Cancer: overview** | 05200_pathways_in_cancer | 0.0037 | Cbx_vs_do_06h |
| **Cancer: overview** | 05204_chemical_carcinogenesis | 0.0367 | Cbx_vs_do_01h |
| **Cancer: overview** | 05205_proteoglycans_in_cancer | 0.0181 | Cbx_vs_do_06h |
| **Cancer: overview** | 05205_proteoglycans_in_cancer | 0.0331 | Control_vs_do_22h |
| **Cancer: overview** | 05206_micrornas_in_cancer | 0.0025 | Cocult_vs_do_06h |
| **Cancer: overview** | 05206_micrornas_in_cancer | 0.0094 | Control_vs_do_22h |
| **Cancer: overview** | 05235_pd-l1_expression_and_pd-1_checkpoint_pathway_in_cancer | 0.0328 | Cbx_vs_do_06h |
| **Cancer: specific types** | 05214_glioma | 0.0347 | Cbx_vs_do_06h |
| **Cancer: specific types** | 05214_glioma | 0.0085 | Control_vs_do_22h |
| **Cancer: specific types** | 05216_thyroid_cancer | 0.0306 | Control_vs_do_22h |
| **Cancer: specific types** | 05218_melanoma | 0.0016 | Control_vs_do_22h |
| **Cancer: specific types** | 05219_bladder_cancer | 0.0286 | Cbx_vs_do_06h |
| **Cancer: specific types** | 05219_bladder_cancer | 0.0086 | Control_vs_do_22h |
| **Cancer: specific types** | 05220_chronic_myeloid_leukemia | 0.0029 | Control_vs_do_22h |
| **Cancer: specific types** | 05222_small_cell_lung_cancer | 0.0218 | Cbx_vs_do_06h |
| **Cancer: specific types** | 05223_non-small_cell_lung_cancer | 0.0016 | Control_vs_do_22h |
| **Cancer: specific types** | 05224_breast_cancer | 0.0130 | Control_vs_do_22h |
| **Cancer: specific types** | 05226_gastric_cancer | 0.0136 | Control_vs_do_22h |
| **Carbohydrate metabolism** | 00650_butanoate_metabolism | 0.0288 | Cbx_vs_do_06h |
| **Cardiovascular disease** | 05410_hypertrophic_cardiomyopathy | 0.0180 | Cbx_vs_do_06h |
| **Cardiovascular disease** | 05414_dilated_cardiomyopathy | 0.0247 | Cbx_vs_do_06h |
| **Cardiovascular disease** | 05417_lipid_and_atherosclerosis | 0.0227 | Cbx_vs_do_06h |
| **Cardiovascular disease** | 05418_fluid_shear_stress_and_atherosclerosis | 0.0220 | Control_vs_do_22h |
| **Cell growth and death** | 04218_cellular_senescence | 0.0029 | Control_vs_do_22h |
| **Cell motility** | 04810_regulation_of_actin_cytoskeleton | 0.0027 | Cbx_vs_do_06h |
| **Development and regeneration** | 04360_axon_guidance | 0.0379 | Control_vs_do_22h |
| **Development and regeneration** | 04380_osteoclast_differentiation | 0.0206 | Control_vs_do_22h |
| **Digestive system** | 04975_fat_digestion_and_absorption | 0.0489 | Cbx_vs_cocult_01h |
| **Digestive system** | 04979_cholesterol_metabolism | 0.0456 | Cbx_vs_do_06h |
| **Digestive system** | 04979_cholesterol_metabolism | 0.0497 | Cbx_vs_do_22h |
| **Drug resistance: antineoplastic** | 01522_endocrine_resistance | 0.0069 | Control_vs_do_22h |
| **Endocrine system** | 03320_ppar_signaling_pathway | 0.0277 | Cbx_vs_do_06h |
| **Endocrine system** | 04910_insulin_signaling_pathway | 0.0222 | Cbx_vs_do_06h |
| **Endocrine system** | 04915_estrogen_signaling_pathway | 0.0310 | Control_vs_do_22h |
| **Endocrine system** | 04917_prolactin_signaling_pathway | 0.0112 | Control_vs_do_22h |
| **Endocrine system** | 04919_thyroid_hormone_signaling_pathway | 0.0063 | Control_vs_do_22h |
| **Endocrine system** | 04920_adipocytokine_signaling_pathway | 0.0305 | Cbx_vs_do_06h |
| **Endocrine system** | 04920_adipocytokine_signaling_pathway | 0.0254 | Control_vs_cbx_22h |
| **Endocrine system** | 04929_gnrh_secretion | 0.0193 | Control_vs_do_22h |
| **Energy metabolism** | 00920_sulfur_metabolism | 0.0361 | Cbx_vs_do_06h |
| **Folding. Sorting and degradation** | 03050_proteasome | 0.0489 | Cbx_vs_cocult_01h |
| **Folding. Sorting and degradation** | 04130_snare_interactions_in_vesicular_transport | 0.0240 | Control_vs_cbx_22h |
| **Glycan biosynthesis and metabolism** | 00512_mucin_type_o-glycan_biosynthesis | 0.0012 | Cbx_vs_do_22h |
| **Glycan biosynthesis and metabolism** | 00512_mucin_type_o-glycan_biosynthesis | 0.0186 | Control_vs_cbx_22h |
| **Glycan biosynthesis and metabolism** | 00514_other_types_of_o-glycan_biosynthesis | 0.0345 | Cbx_vs_do_22h |
| **Glycan biosynthesis and metabolism** | 00515_mannose_type_o-glycan_biosynthesis | 0.0009 | Control_vs_do_22h |
| **Immune disease** | 05323_rheumatoid_arthritis | 0.0026 | Cbx_vs_do_06h |
| **Immune disease** | 05340_primary_immunodeficiency | 0.0161 | Cbx_vs_do_06h |
| **Immune system** | 04062_chemokine_signaling_pathway | 0.0152 | Cbx_vs_cocult_01h |
| **Immune system** | 04062_chemokine_signaling_pathway | 0.0024 | Control_vs_do_22h |
| **Immune system** | 04620_toll-like_receptor_signaling_pathway | 0.0274 | Cbx_vs_do_22h |
| **Immune system** | 04640_hematopoietic_cell_lineage | 0.0459 | Control_vs_do_22h |
| **Immune system** | 04658_th1_and_th2_cell_differentiation | 0.0067 | Control_vs_cbx_22h |
| **Immune system** | 04659_th17_cell_differentiation | 0.0243 | Control_vs_cbx_22h |
| **Immune system** | 04672_intestinal_immune_network_for_iga_production | 0.0462 | Cocult_vs_do_06h |
| **Immune system** | 04672_intestinal_immune_network_for_iga_production | 0.0220 | Control_vs_do_22h |
| **Infectious disease: bacterial** | 05135_yersinia_infection | 0.0361 | Cbx_vs_do_06h |
| **Infectious disease: bacterial** | 05152_tuberculosis | 0.0379 | Cbx_vs_do_06h |
| **Infectious disease: viral** | 05160_hepatitis_c | 0.0142 | Control_vs_do_22h |
| **Infectious disease: viral** | 05163_human_cytomegalovirus_infection | 0.0008 | Control_vs_do_22h |
| **Infectious disease: viral** | 05167_kaposi_sarcoma-associated_herpesvirus_infection | 0.0244 | Control_vs_do_22h |
| **Infectious disease: viral** | 05169_epstein-barr_virus_infection | 0.0059 | Control_vs_do_22h |
| **Infectious disease: viral** | 05170_human_immunodeficiency_virus_1_infection | 0.0244 | Cbx_vs_do_06h |
| **Infectious disease: viral** | 05170_human_immunodeficiency_virus_1_infection | 0.0162 | Control_vs_do_22h |
| **Nervous system** | 04721_synaptic_vesicle_cycle | 0.0157 | Control_vs_cbx_22h |
| **Nervous system** | 04722_neurotrophin_signaling_pathway | 0.0244 | Control_vs_do_22h |
| **Nervous system** | 04723_retrograde_endocannabinoid_signaling | 0.0387 | Control_vs_do_22h |
| **Nervous system** | 04726_serotonergic_synapse | 0.0374 | Control_vs_do_22h |
| **Replication and repair** | 03030_dna_replication | 0.0011 | Cocult_vs_do_06h |
| **Replication and repair** | 03420_nucleotide_excision_repair | 0.0266 | Cocult_vs_do_06h |
| **Replication and repair** | 03430_mismatch_repair | 0.0074 | Cocult_vs_do_06h |
| **Sensory system** | 04740_olfactory_transduction | 0.0198 | Cbx_vs_do_06h |
| **Signal transduction** | 04010_mapk_signaling_pathway | 0.0091 | Control_vs_do_22h |
| **Signal transduction** | 04014_ras_signaling_pathway | 0.0041 | Cbx_vs_do_06h |
| **Signal transduction** | 04015_rap1_signaling_pathway | 0.0116 | Cbx_vs_do_06h |
| **Signal transduction** | 04020_calcium_signaling_pathway | 0.0141 | Cbx_vs_do_06h |
| **Signal transduction** | 04064_nf-kappa_b_signaling_pathway | 0.0056 | Cbx_vs_cocult_01h |
| **Signal transduction** | 04064_nf-kappa_b_signaling_pathway | 0.0089 | Cbx_vs_do_06h |
| **Signal transduction** | 04068_foxo_signaling_pathway | 0.0396 | Cbx_vs_do_06h |
| **Signal transduction** | 04070_phosphatidylinositol_signaling_system | 0.0146 | Cbx_vs_do_01h |
| **Signal transduction** | 04070_phosphatidylinositol_signaling_system | 0.0475 | Control_vs_cbx_22h |
| **Signal transduction** | 04152_ampk_signaling_pathway | 0.0361 | Cbx_vs_do_06h |
| **Signal transduction** | 04330_notch_signaling_pathway | 0.0415 | Control_vs_cbx_22h |
| **Signal transduction** | 04371_apelin_signaling_pathway | 0.0010 | Cbx_vs_do_06h |
| **Signaling molecules and interaction** | 04060_cytokine-cytokine_receptor_interaction | 0.0168 | Control_vs_do_22h |
| **Signaling molecules and interaction** | 04061_viral_protein_interaction_with_cytokine  _and_cytokine_receptor | 0.0235 | Control_vs_do_22h |
| **Signaling molecules and interaction** | 04514_cell_adhesion_molecules | 0.0320 | Control_vs_cbx_22h |
| **Substance dependence** | 05032_morphine_addiction | 0.0212 | Control_vs_do_22h |
| **Transport and catabolism** | 04140_autophagy | 0.0091 | Cbx_vs_cocult_01h |
| **Transport and catabolism** | 04144_endocytosis | 0.0194 | Control_vs_do_22h |
| **Xenobiotics biodegradation and metabolism** | 00980_metabolism_of_xenobiotics_by_cytochrome_p450 | 0.0394 | Cbx_vs_do_01h |
| **Xenobiotics biodegradation and metabolism** | 00982_drug_metabolism | 0.0327 | Cbx_vs_do_01h |

Table S9: The 116 GO-terms from the biological processes that were over-represented with their indicative p-values.

| GO-term | P-value |
| --- | --- |
| GO:0009992_cellular_water_homeostasis | 0.001587242 |
| GO:0032814_regulation_of_natural_killer_cell_activation | 0.001587242 |
| GO:0014068_positive_regulation_of_phosphatidylinositol_3-kinase_signaling | 0.001818883 |
| GO:0043547_positive_regulation_of_gtpase_activity | 0.002710932 |
| GO:0016310_phosphorylation | 0.003641162 |
| GO:0051148_negative_regulation_of_muscle_cell_differentiation | 0.004635574 |
| GO:0015696_ammonium_transport | 0.009026351 |
| GO:0016358_dendrite_development | 0.009026351 |
| GO:0046856_phosphatidylinositol_dephosphorylation | 0.009026351 |
| GO:0050921_positive_regulation_of_chemotaxis | 0.009026351 |
| GO:0071300_cellular_response_to_retinoic_acid | 0.009026351 |
| GO:2000251_positive_regulation_of_actin_cytoskeleton_reorganization | 0.009026351 |
| GO:0006833_water_transport | 0.02139584 |
| GO:0050765_negative_regulation_of_phagocytosis | 0.02139584 |
| GO:1903076_regulation_of_protein_localization_to_plasma_membrane | 0.02139584 |
| GO:0032868_response_to_insulin | 0.029171326 |
| GO:0042060_wound_healing | 0.029171326 |
| GO:0051894_positive_regulation_of_focal_adhesion_assembly | 0.029171326 |
| GO:0043087_regulation_of_gtpase_activity | 0.03788211 |
| GO:0048146_positive_regulation_of_fibroblast_proliferation | 0.03788211 |
| GO:0001765_membrane_raft_assembly | 0.039873861 |
| GO:0001768_establishment_of_t_cell_polarity | 0.039873861 |
| GO:0001936_regulation_of_endothelial_cell_proliferation | 0.039873861 |
| GO:0002127_trna_wobble_base_cytosine_methylation | 0.039873861 |
| GO:0002264_endothelial_cell_activation_involved_in_immune_response | 0.039873861 |
| GO:0003181_atrioventricular_valve_morphogenesis | 0.039873861 |
| GO:0003208_cardiac_ventricle_morphogenesis | 0.039873861 |
| GO:0003300_cardiac_muscle_hypertrophy | 0.039873861 |
| GO:0006568_tryptophan_metabolic_process | 0.039873861 |
| GO:0006884_cell_volume_homeostasis | 0.039873861 |
| GO:0006900_vesicle_budding_from_membrane | 0.039873861 |
| GO:0006903_vesicle_targeting | 0.039873861 |
| GO:0006904_vesicle_docking_involved_in_exocytosis | 0.039873861 |
| GO:0010038_response_to_metal_ion | 0.039873861 |
| GO:0010894_negative_regulation_of_steroid_biosynthetic_process | 0.039873861 |
| GO:0014029_neural_crest_formation | 0.039873861 |
| GO:0014031_mesenchymal_cell_development | 0.039873861 |
| GO:0015670_carbon_dioxide_transport | 0.039873861 |
| GO:0016255_attachment_of_gpi_anchor_to_protein | 0.039873861 |
| GO:0017196_n-terminal_peptidyl-methionine_acetylation | 0.039873861 |
| GO:0018277_protein_deamination | 0.039873861 |
| GO:0030104_water_homeostasis | 0.039873861 |
| GO:0030575_nuclear_body_organization | 0.039873861 |
| GO:0030950_establishment_or_maintenance_of_actin_cytoskeleton_polarity | 0.039873861 |
| GO:0032049_cardiolipin_biosynthetic_process | 0.039873861 |
| GO:0032817_regulation_of_natural_killer_cell_proliferation | 0.039873861 |
| GO:0032878_regulation_of_establishment_or_maintenance_of_cell_polarity | 0.039873861 |
| GO:0033292_t-tubule_organization | 0.039873861 |
| GO:0033363_secretory_granule_organization | 0.039873861 |
| GO:0034112_positive_regulation_of_homotypic_cell-cell_adhesion | 0.039873861 |
| GO:0034219_carbohydrate_transmembrane_transport | 0.039873861 |
| GO:0035021_negative_regulation_of_rac_protein_signal_transduction | 0.039873861 |
| GO:0035378_carbon_dioxide_transmembrane_transport | 0.039873861 |
| GO:0038195_urokinase_plasminogen_activator_signaling_pathway | 0.039873861 |
| GO:0042269_regulation_of_natural_killer_cell_mediated_cytotoxicity | 0.039873861 |
| GO:0042420_dopamine_catabolic_process | 0.039873861 |
| GO:0042998_positive_regulation_of_golgi_to_plasma_membrane_protein_transport | 0.039873861 |
| GO:0043587_tongue_morphogenesis | 0.039873861 |
| GO:0043620_regulation_of_dna-templated_transcription_in_response_to_stress | 0.039873861 |
| GO:0044088_regulation_of_vacuole_organization | 0.039873861 |
| GO:0044241_lipid_digestion | 0.039873861 |
| GO:0046325_negative_regulation_of_glucose_import | 0.039873861 |
| GO:0046466_membrane_lipid_catabolic_process | 0.039873861 |
| GO:0046878_positive_regulation_of_saliva_secretion | 0.039873861 |
| GO:0048208_copii_vesicle_coating | 0.039873861 |
| GO:0048387_negative_regulation_of_retinoic_acid_receptor_signaling_pathway | 0.039873861 |
| GO:0048593_camera-type_eye_morphogenesis | 0.039873861 |
| GO:0050999_regulation_of_nitric-oxide_synthase_activity | 0.039873861 |
| GO:0051057_positive_regulation_of_small_gtpase_mediated_signal_transduction | 0.039873861 |
| GO:0051099_positive_regulation_of_binding | 0.039873861 |
| GO:0051458_corticotropin_secretion | 0.039873861 |
| GO:0051591_response_to_camp | 0.039873861 |
| GO:0051620_norepinephrine_uptake | 0.039873861 |
| GO:0051697_protein_delipidation | 0.039873861 |
| GO:0055003_cardiac_myofibril_assembly | 0.039873861 |
| GO:0060428_lung_epithelium_development | 0.039873861 |
| GO:0060669_embryonic_placenta_morphogenesis | 0.039873861 |
| GO:0061484_hematopoietic_stem_cell_homeostasis | 0.039873861 |
| GO:0070661_leukocyte_proliferation | 0.039873861 |
| GO:0070862_negative_regulation_of_protein_exit_from_endoplasmic_reticulum | 0.039873861 |
| GO:0070901_mitochondrial_trna_methylation | 0.039873861 |
| GO:0070925_organelle_assembly | 0.039873861 |
| GO:0071241_cellular_response_to_inorganic_substance | 0.039873861 |
| GO:0071472_cellular_response_to_salt_stress | 0.039873861 |
| GO:0071786_endoplasmic_reticulum_tubular_network_organization | 0.039873861 |
| GO:0072014_proximal_tubule_development | 0.039873861 |
| GO:0072220_metanephric_descending_thin_limb_development | 0.039873861 |
| GO:0072230_metanephric_proximal_straight_tubule_development | 0.039873861 |
| GO:0072232_metanephric_proximal_convoluted_tubule_segment_2_development | 0.039873861 |
| GO:0086014_atrial_cardiac_muscle_cell_action_potential | 0.039873861 |
| GO:0086015_sa_node_cell_action_potential | 0.039873861 |
| GO:0090217_negative_regulation_of_1-phosphatidylinositol-4-phosphate_5-kinase_activity | 0.039873861 |
| GO:0097029_mature_conventional_dendritic_cell_differentiation | 0.039873861 |
| GO:0098781_ncrna_transcription | 0.039873861 |
| GO:0098868_bone_growth | 0.039873861 |
| GO:0098914_membrane_repolarization_during_atrial_cardiac_muscle_cell_action_potential | 0.039873861 |
| GO:1900020_positive_regulation_of_protein_kinase_c_activity | 0.039873861 |
| GO:1900135_positive_regulation_of_renin_secretion_into_blood_stream | 0.039873861 |
| GO:1901303_negative_regulation_of_cargo_loading_into_copii-coated_vesicle | 0.039873861 |
| GO:1902033_regulation_of_hematopoietic_stem_cell_proliferation | 0.039873861 |
| GO:1902605_heterotrimeric_g-protein_complex_assembly | 0.039873861 |
| GO:1902635_1-phosphatidyl-1d-myo-inositol_4.5-bisphosphate_biosynthetic_process | 0.039873861 |
| GO:1902685_positive_regulation_of_receptor_localization_to_synapse | 0.039873861 |
| GO:1903307_positive_regulation_of_regulated_secretory_pathway | 0.039873861 |
| GO:1903363_negative_regulation_of_cellular_protein_catabolic_process | 0.039873861 |
| GO:1904015_cellular_response_to_serotonin | 0.039873861 |
| GO:1905062_positive_regulation_of_cardioblast_proliferation | 0.039873861 |
| GO:1905702_regulation_of_inhibitory_synapse_assembly | 0.039873861 |
| GO:1990748_cellular_detoxification | 0.039873861 |
| GO:2000341_regulation_of_chemokine_(c-x-c_motif)_ligand_2_production | 0.039873861 |
| GO:2000425_regulation_of_apoptotic_cell_clearance | 0.039873861 |
| GO:2000640_positive_regulation_of_srebp_signaling_pathway | 0.039873861 |
| GO:2000786_positive_regulation_of_autophaGOsome_assembly | 0.039873861 |
| GO:2001037_positive_regulation_of_tongue_muscle_cell_differentiation | 0.039873861 |
| GO:0006888_endoplasmic_reticulum_to_golgi_vesicle-mediated_transport | 0.047441513 |
| GO:0090200_positive_regulation_of_release_of_cytochrome_c_from_mitochondria | 0.047441513 |

Table S10: The 11 GO-terms from the cellular component that were over-represented with their indicative p-values.

| GO-term | P-value |
| --- | --- |
| GO:0042765_gpi-anchor_transamidase_complex | 0.001587242 |
| GO:0071565_nbaf_complex | 0.01464804 |
| GO:0080008_cul4-ring_e3_ubiquitin_ligase_complex | 0.01464804 |
| GO:0009368_endopeptidase_clp_complex | 0.039873861 |
| GO:0030118_clathrin_coat | 0.039873861 |
| GO:0032127_dense_core_granule_membrane | 0.039873861 |
| GO:0034066_ric1-rgp1_guanyl-nucleotide_exchange_factor_complex | 0.039873861 |
| GO:0042582_azurophil_granule | 0.039873861 |
| GO:0070820_tertiary_granule | 0.039873861 |
| GO:0097450_astrocyte_end-foot | 0.039873861 |
| GO:1990665_anxa2-p11_complex | 0.039873861 |

Table S11: The 37 GO-terms from the molecular function that were over-represented with their indicative p-values.

| GO-Term | P-value |
| --- | --- |
| GO:0015250_water_channel_activity | 0.01464804 |
| GO:0003720_telomerase_activity | 0.039873861 |
| GO:0003721_telomerase_rna_reverse_transcriptase_activity | 0.039873861 |
| GO:0003923_gpi-anchor_transamidase_activity | 0.039873861 |
| GO:0004112_cyclic-nucleotide_phosphodiesterase_activity | 0.039873861 |
| GO:0004500_dopamine_beta-monooxygenase_activity | 0.039873861 |
| GO:0005223_intracellular_cgmp-activated_cation_channel_activity | 0.039873861 |
| GO:0005328_neurotransmitter:sodium_symporter_activity | 0.039873861 |
| GO:0008401_retinoic_acid_4-hydroxylase_activity | 0.039873861 |
| GO:0015144_carbohydrate_transmembrane_transporter_activity | 0.039873861 |
| GO:0016213_linoleoyl-coa_desaturase_activity | 0.039873861 |
| GO:0016309_1-phosphatidylinositol-5-phosphate_4-kinase_activity | 0.039873861 |
| GO:0016524_latrotoxin_receptor_activity | 0.039873861 |
| GO:0017060_3-galactosyl-n-acetylglucosaminide_4-alpha-l-fucosyltransferase_activity | 0.039873861 |
| GO:0019215_intermediate_filament_binding | 0.039873861 |
| GO:0019958_c-x-c_chemokine_binding | 0.039873861 |
| GO:0030184_nitric_oxide_transmembrane_transporter_activity | 0.039873861 |
| GO:0030292_protein_tyrosine_kinase_inhibitor_activity | 0.039873861 |
| GO:0030377_urokinase_plasminogen_activator_receptor_activity | 0.039873861 |
| GO:0031433_telethonin_binding | 0.039873861 |
| GO:0035325_toll-like_receptor_binding | 0.039873861 |
| GO:0035379_carbon_dioxide_transmembrane_transporter_activity | 0.039873861 |
| GO:0047408_alkenylglycerophosphocholine_hydrolase_activity | 0.039873861 |
| GO:0047409_alkenylglycerophosphoethanolamine_hydrolase_activity | 0.039873861 |
| GO:0050568_protein-glutamine_glutaminase_activity | 0.039873861 |
| GO:0050683_af-1_domain_binding | 0.039873861 |
| GO:0052906_trna_(guanine(37)-n(1))-methyltransferase_activity | 0.039873861 |
| GO:0070287_ferritin_receptor_activity | 0.039873861 |
| GO:0086089_voltage-gated_potassium_channel_activity_involved_in_atrial_cardiac_muscle_cell_action_potential_repolarization | 0.039873861 |
| GO:0103046_alanylglutamate_dipeptidase_activity | 0.039873861 |
| GO:0120294_peptide_serotonyltransferase_activity | 0.039873861 |
| GO:0120295_histone_serotonyltransferase_activity | 0.039873861 |
| GO:0120297_histone_dopaminyltransferase_activity | 0.039873861 |
| GO:0120298_peptide_noradrenalinyltransferase_activity | 0.039873861 |
| GO:0120299_peptide_histaminyltransferase_activity | 0.039873861 |
| GO:1990782_protein_tyrosine_kinase_binding | 0.039873861 |
| GO:1990931_mrna_n6-methyladenosine_dioxygenase_activity | 0.039873861 |

**Table S12**: The used primers for RT-qPCR

| **Target miRNA** | **Accession** | **Sequence** | **Qiagen GG cat no:** |
| --- | --- | --- | --- |
| miR-21-5p | MIMAT0000076 | 5'UAGCUUAUCAGACUGAUGUUGA | YP00204230 |
| miR-92a | MIMAT0000092 | 5'UAUUGCACUUGUCCCGGCCUGU | YP00204258 |
| miR-93 | MIMAT0003837 | 5'CAAAGUGCUGUUCGUGCAGGUA | YP02117906 |
| miR-127 | MIMAT0000446 | 5'UCGGAUCCGUCUGAGCUUGGCU | YP00204048 |

# Supplementary Figures


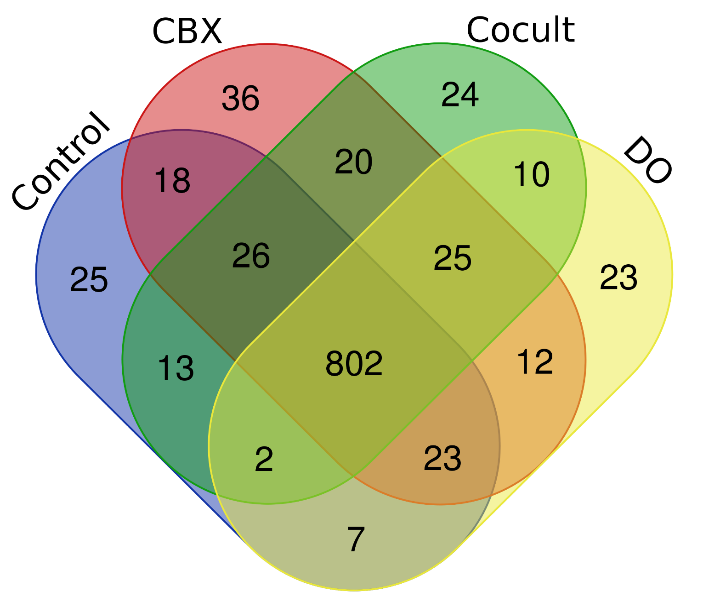


Figure S1: A Venn diagram illustrating the number of miRNAs present in each group of oocytes after one hour of maturation


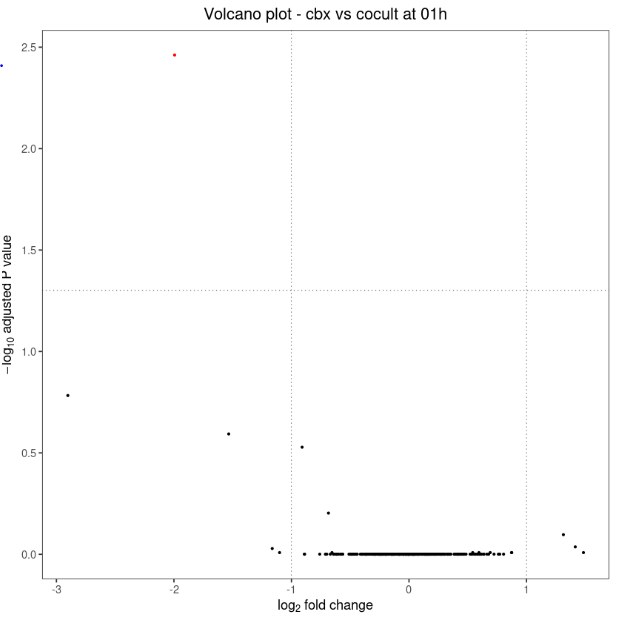


Figure S2: The differentially expressed miRNA between the CBX group and the co-culturing group after one hour of maturation is shown in a Volcano plot. One statistically significant result (PAdj < 0.05 and |log2FC| ≥ 1) (red dot) was up-regulated in the CBX group.


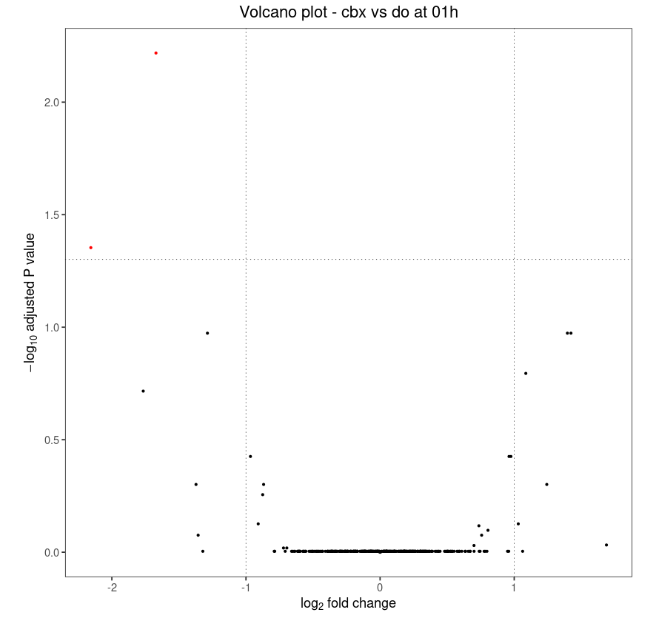


Figure S3: The differentially expressed miRNAs between CBX group and the DO group after one hour of maturation are shown in a Volcano plot. Two statistically significant results (PAdj < 0.05 and |log2FC|≥1) (red dots) were found. both up-regulated in the CBX group.


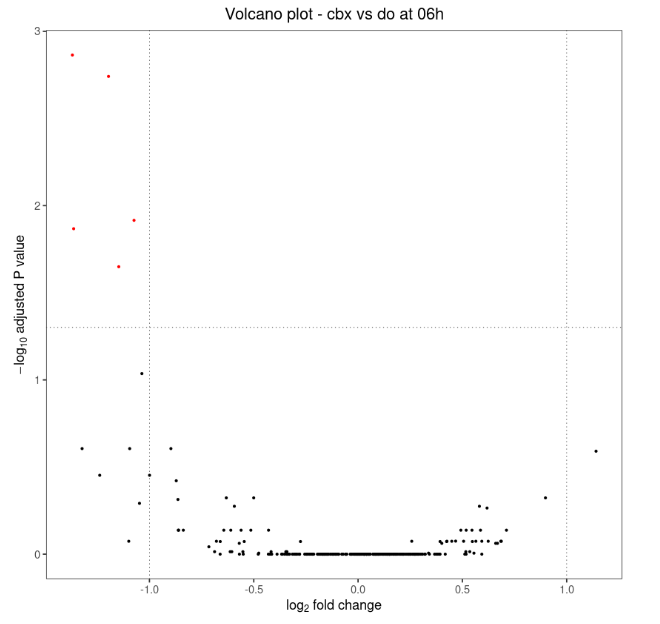


Figure S4: The differentially expressed miRNAs between CBX group and the DO group after six hours of maturation are shown in a Volcano plot. Five statistically significant results (PAdj < 0.05 and |log2FC|≥1) (red dots) were found, all up-regulated in the CBX group.


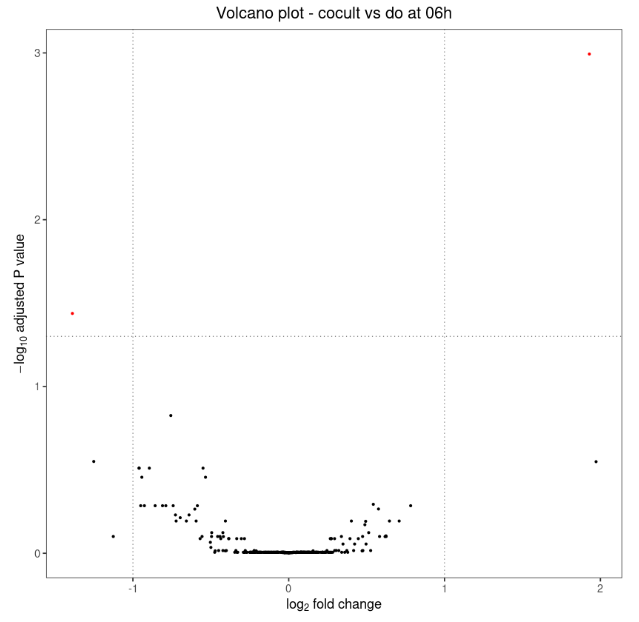


Figure S5: The differentially expressed miRNAs between the co-culture group and the DO group after six hours of maturation are shown in a Volcano plot. Two statistically significant results (PAdj 0.05 and |log2FC|≥1) (red dots) were found. one up-regulated, and one down-regulated in the DO group.


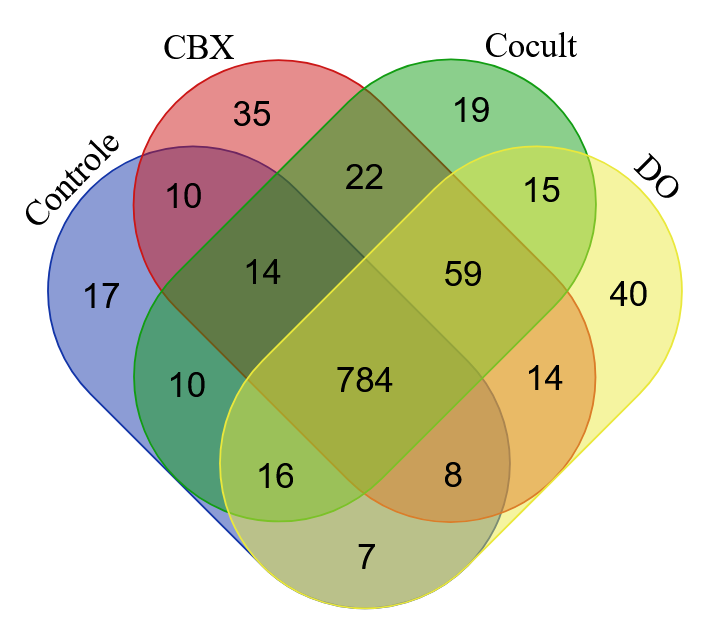


Figure S6: A Venn diagram illustrating the number of miRNAs present in each group of oocytes after six hours of maturation


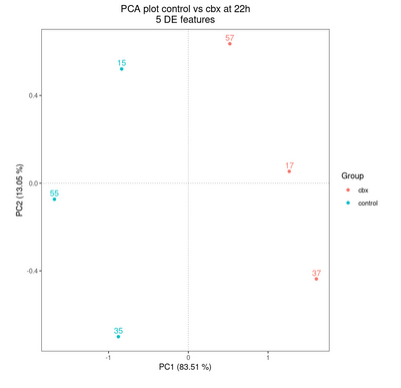


Figure S7: The PCA plot from the five differentially expressed miRNAs between the control group and the CBX group after 22 hours of maturation.


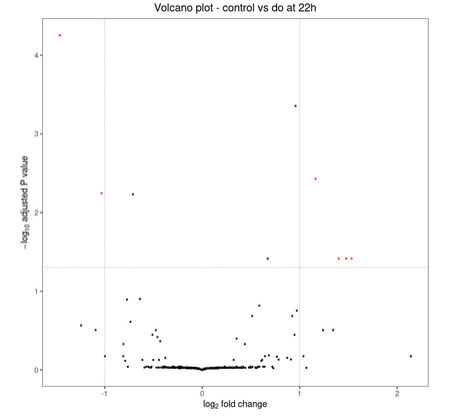


Figure S8: The differentially expressed miRNAs between the CBX group and the control group after 22 hours of maturation are shown in a Volcano plot. Two statistically significant results (PAdj < 0.05 and |log2FC|≥1) (red dot) were found, four up-regulated and two down-regulated in the DO group.


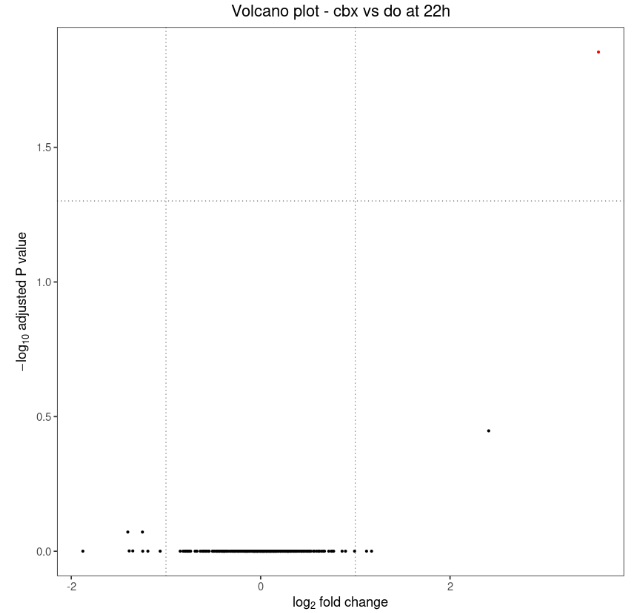


Figure S9: The differentially expressed miRNA between the CBX group and the DO group after 22 hours of maturation is shown in a Volcano plot. One statistically significant result (PAdj < 0.05 and |log2FC|≥1) (red dot) was found, up-regulated in the DO group.


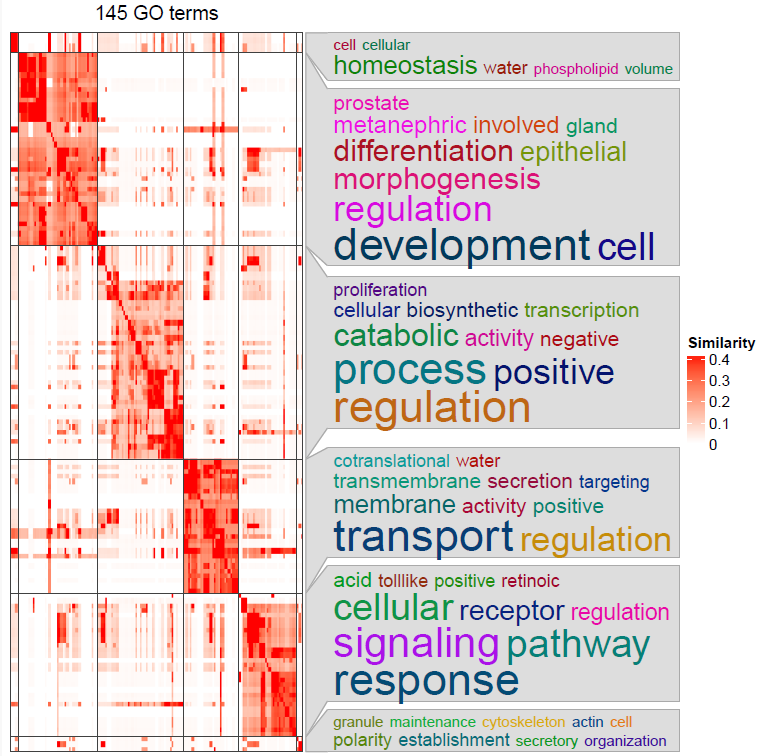


Figure S10: The heatmap corresponding to the 145 over-represented GO-terms from biological process for the comparison between control and DO after 22 hours of maturation, with a color gradient from white to red for similarity between 0 – 0.4.


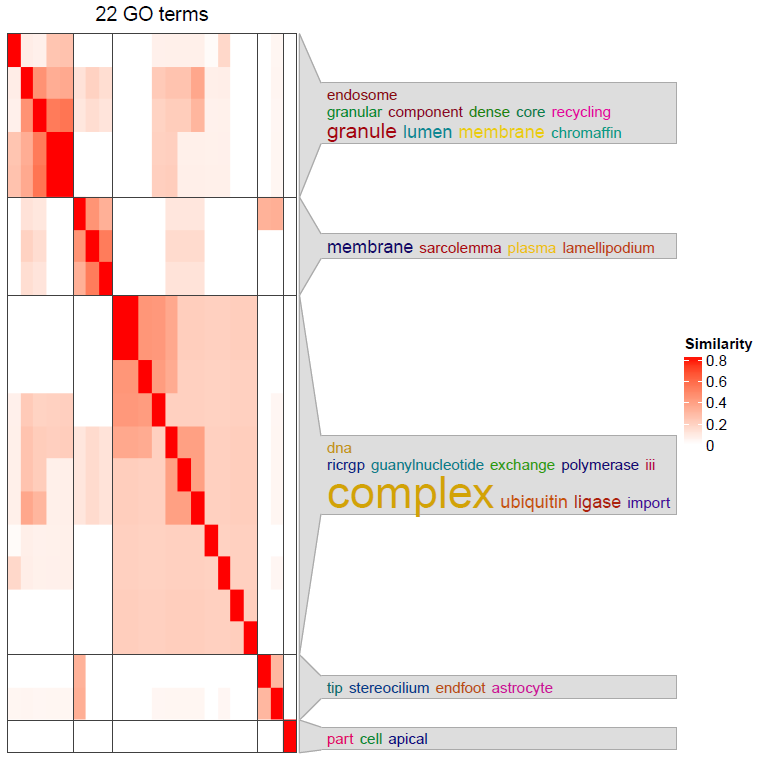


Figure S11: The heatmap corresponding to the 22 over-represented GO-terms cellular components for the comparison between control and DO after 22 hours of maturation, with a color gradient from white to red for similarity between 0 – 0.8.


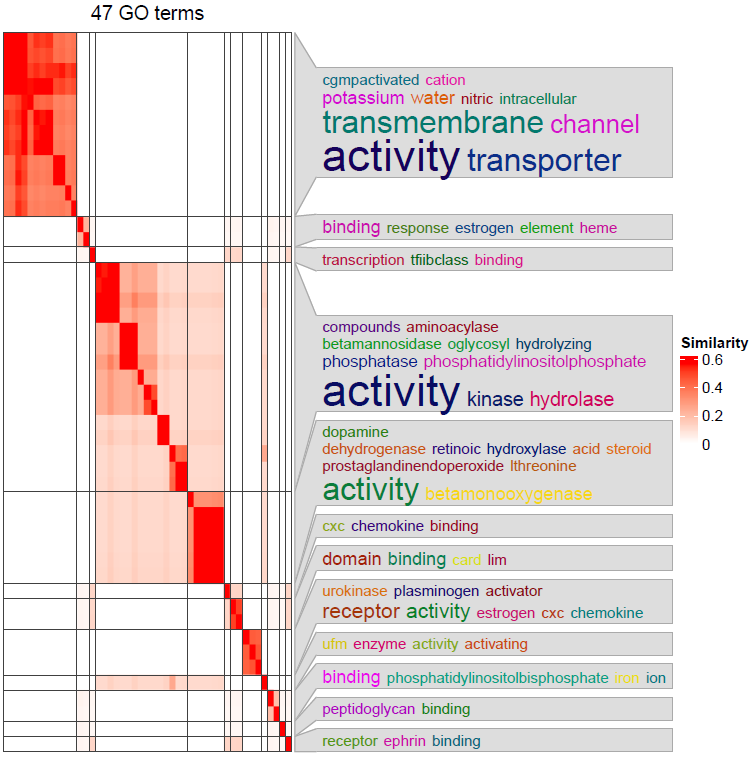


Figure S12: The heatmap corresponding to the 47 over-represented GO-terms molecular function for the comparison between control and DO after 22 hours of maturation, with a color gradient from white to red for similarity between 0 – 0.8.


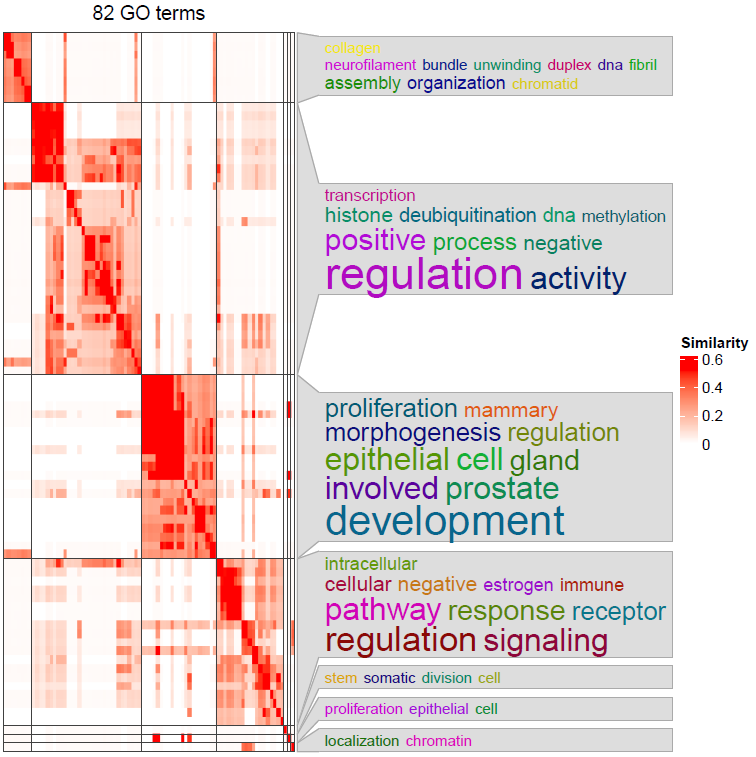


Figure S13: The heatmap corresponding to the 82 over-represented GO-terms biological process for the comparison between co-culture and DO after 6 hours of maturation, with a color gradient from white to red for similarity between 0 – 0.6.


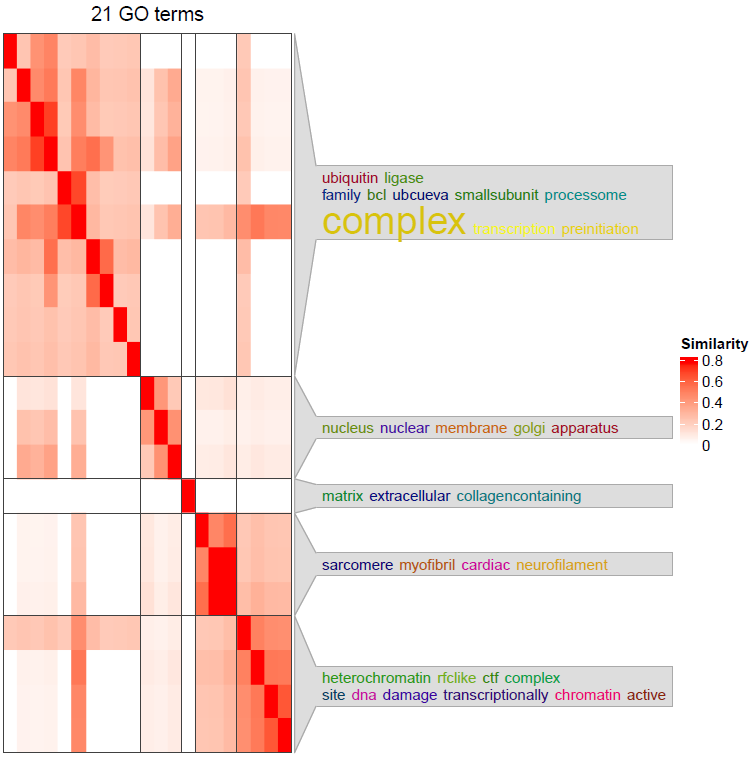


Figure S14: The heatmap corresponding to the 21 over-represented GO-terms cellular component for the comparison between co-culture and DO after 6 hours of maturation, with a color gradient from white to red for similarity between 0 – 0.8.


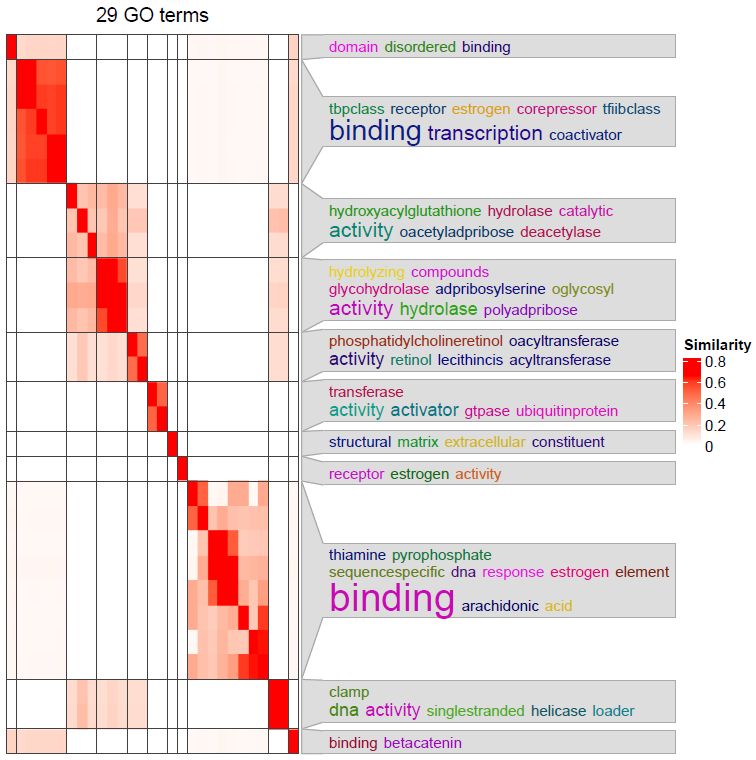


Figure S15: The heatmap corresponding to the 29 over-represented GO-terms molecular function for the comparison between co-culture and DO after 6 hours of maturation, with a color gradient from white to red for similarity between 0 – 0.8.


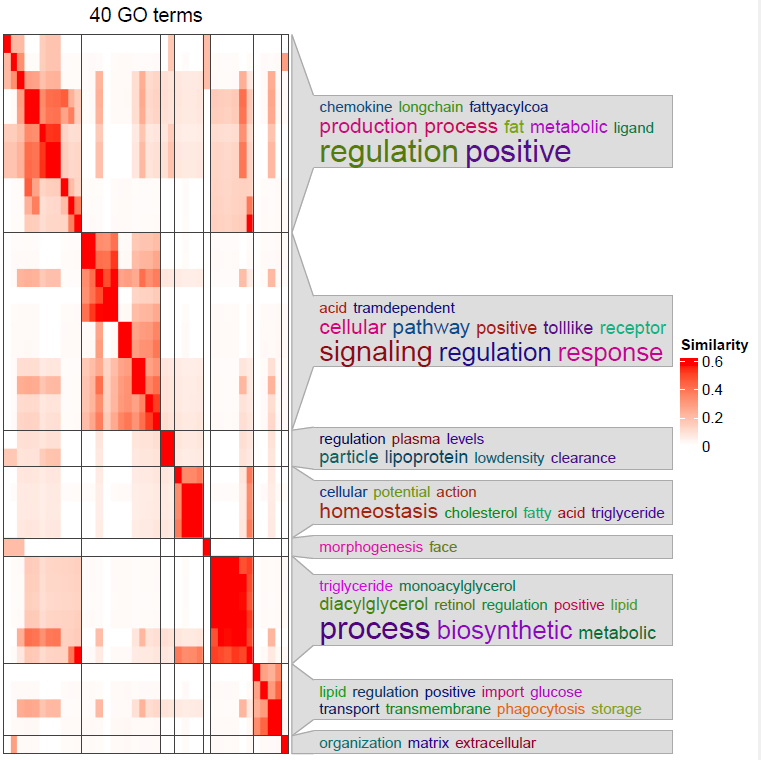


**Figure S16**: The heatmap corresponding to the 40 over-represented GO-terms biological process for the comparison between co-culture and CBX after 1 hour of maturation, with a color gradient from white to red for similarity between 0 – 0.6.


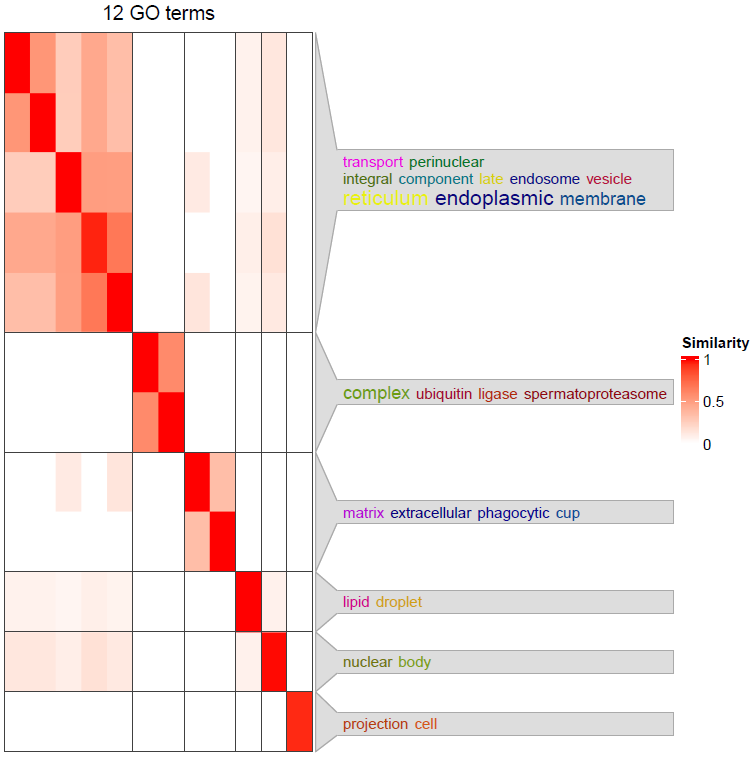


**Figure S17**: The heatmap corresponding to the 12 over-represented GO-terms cellular component for the comparison between co-culture and CBX after 1 hour of maturation, with a color gradient from white to red for similarity between 0 – 1.


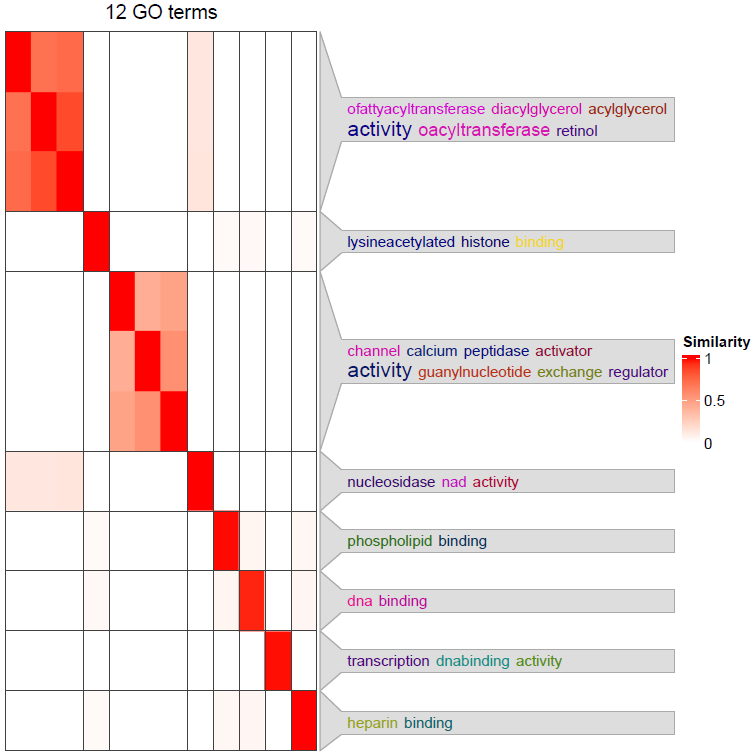


**Figure S18**: The heatmap corresponding to the 12 over-represented GO-terms molecular function for the comparison between co-culture and CBX after 1 hour of maturation, with a color gradient from white to red for similarity between 0 – 1.


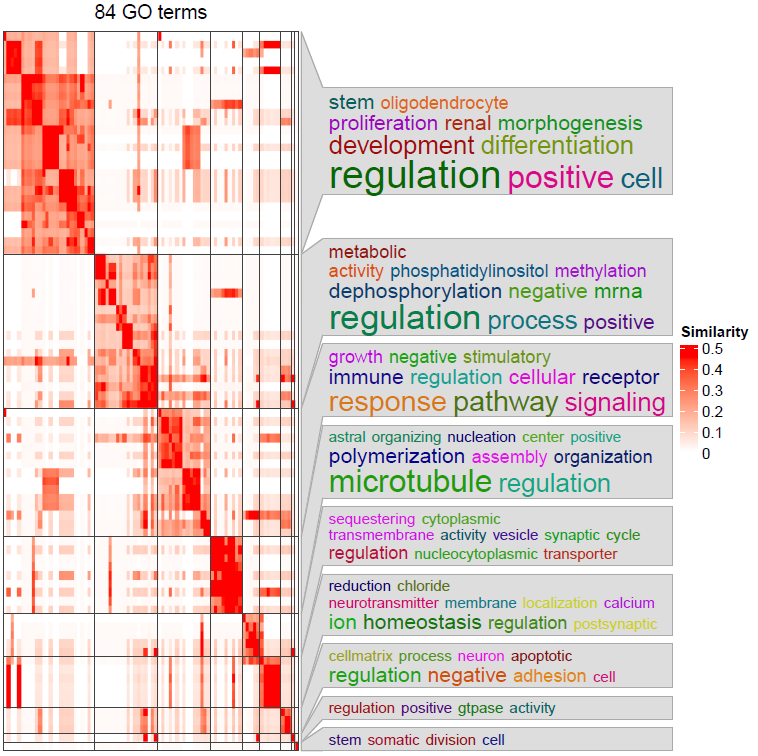


**Figure S19**: The heatmap corresponding to the 84 over-represented GO-terms biological process for the comparison between DO and CBX after 1 hour of maturation, with a color gradient from white to red for similarity between 0 – 0.5.


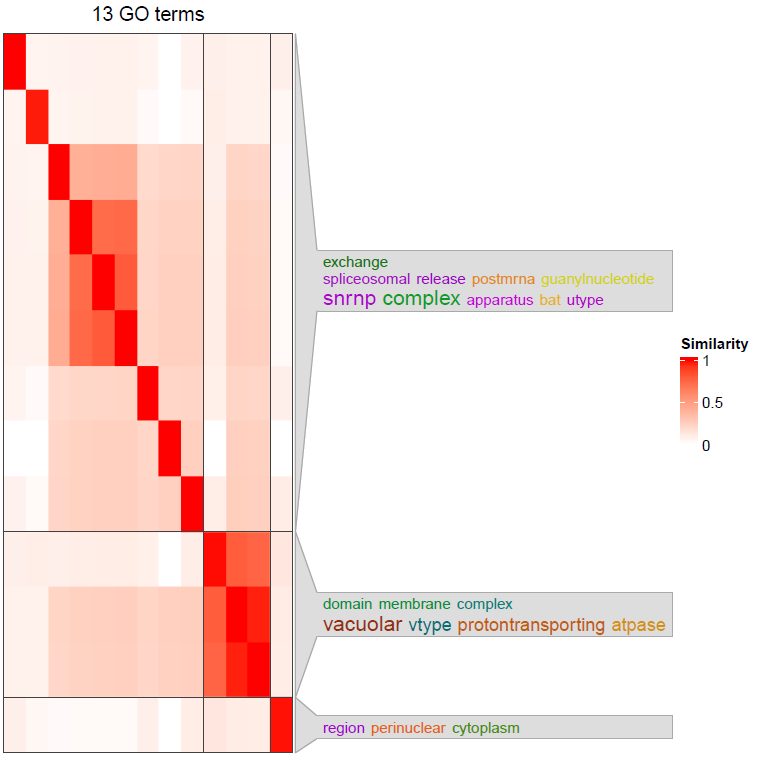


**Figure S20**: The heatmap corresponding to the 13 over-represented GO-terms cellular component for the comparison between DO and CBX after 1 hour of maturation, with a color gradient from white to red for similarity between 0 – 1.


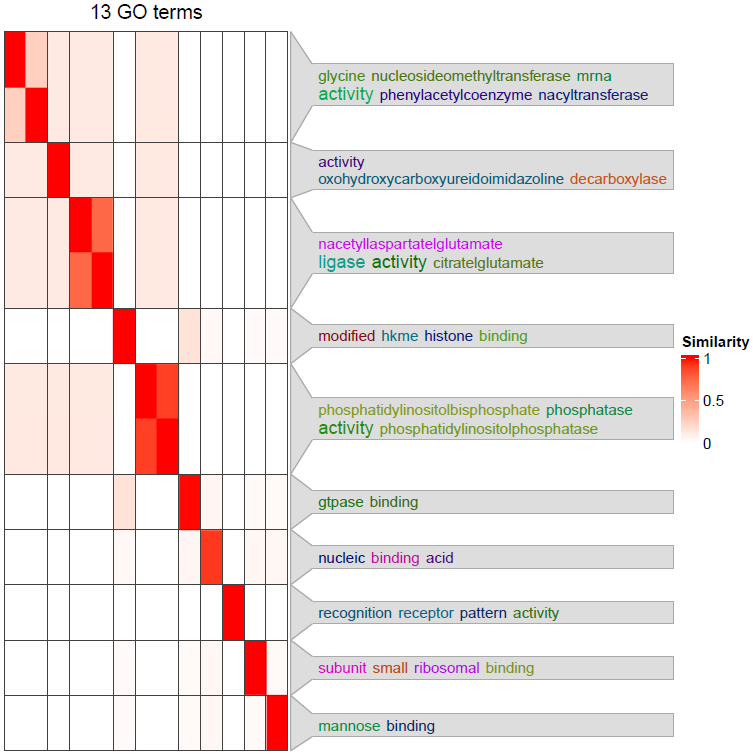


**Figure S21**: The heatmap corresponding to the 13 over-represented GO-terms molecular function for the comparison between DO and CBX after 1 hour of maturation, with a color gradient from white to red for similarity between 0 – 1.


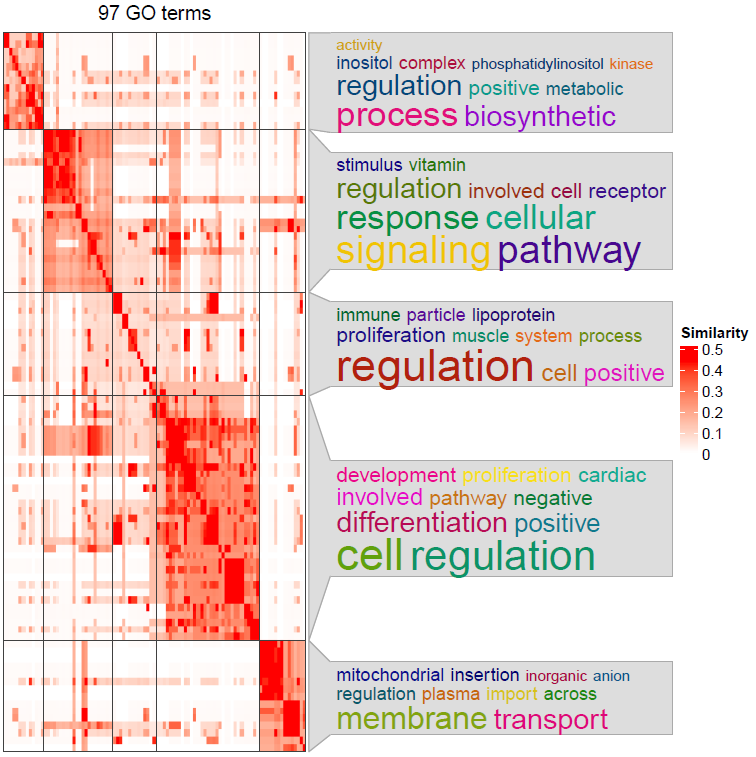


**Figure S22**: The heatmap corresponding to the 97 over-represented GO-terms biological process for the comparison between DO and CBX after 6 hours of maturation, with a color gradient from white to red for similarity between 0 – 0.5.


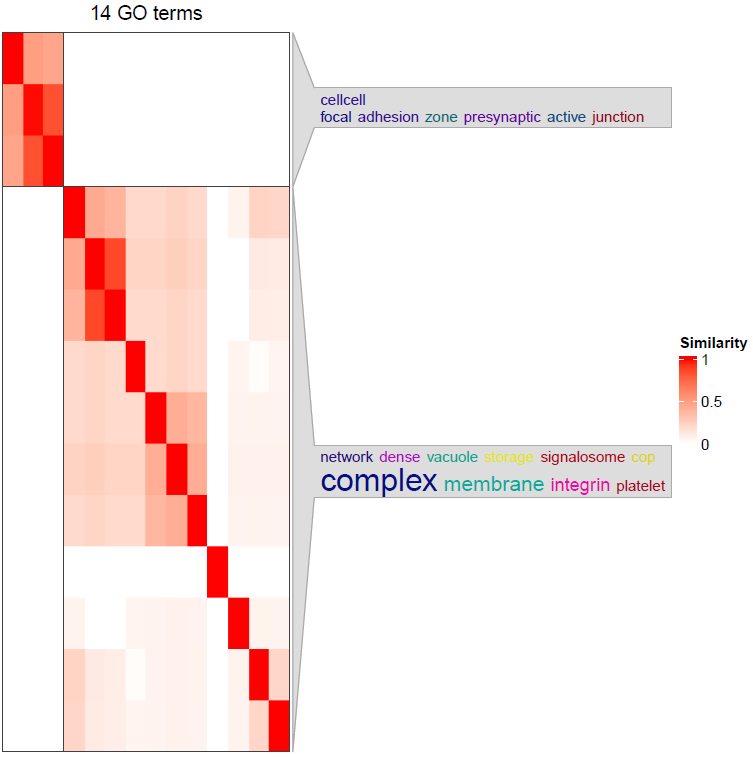


**Figure S23**: The heatmap corresponding to the 14 over-represented GO-terms cellular component for the comparison between DO and CBX after 6 hours of maturation, with a color gradient from white to red for similarity between 0 – 1.


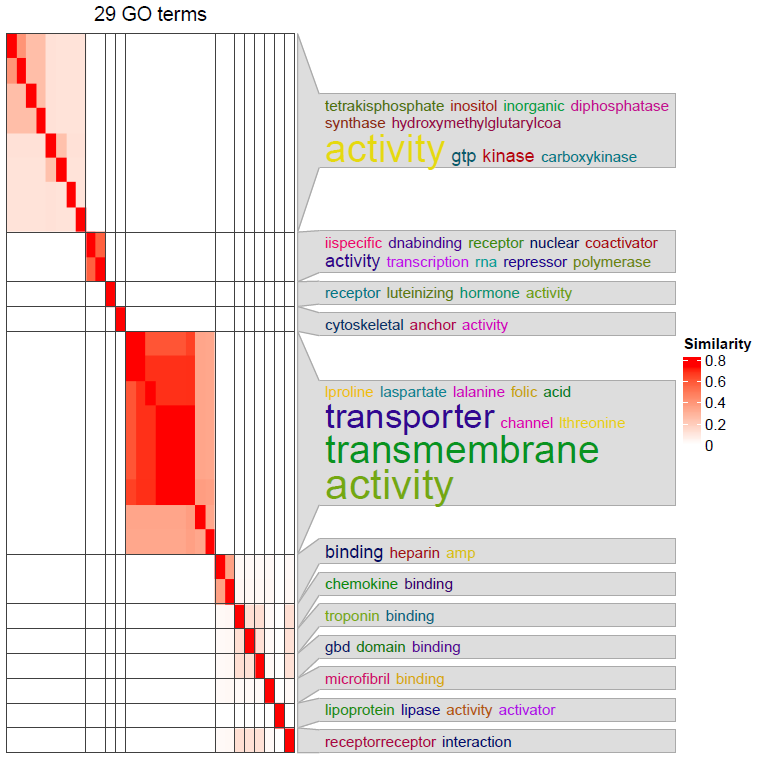


**Figure S24**: The heatmap corresponding to the 29 over-represented GO-terms molecular function for the comparison between DO and CBX after 6 hours of maturation, with a color gradient from white to red for similarity between 0 – 0.8.


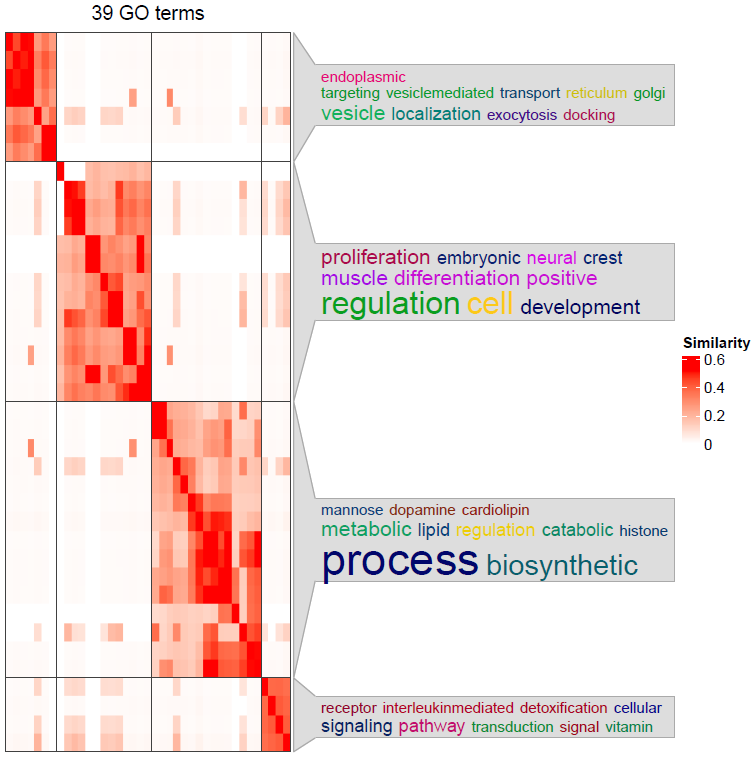


**Figure S25**: The heatmap corresponding to the 39 over-represented GO-terms biological process for the comparison between DO and CBX after 22 hours of maturation, with a color gradient from white to red for similarity between 0 – 0.6.


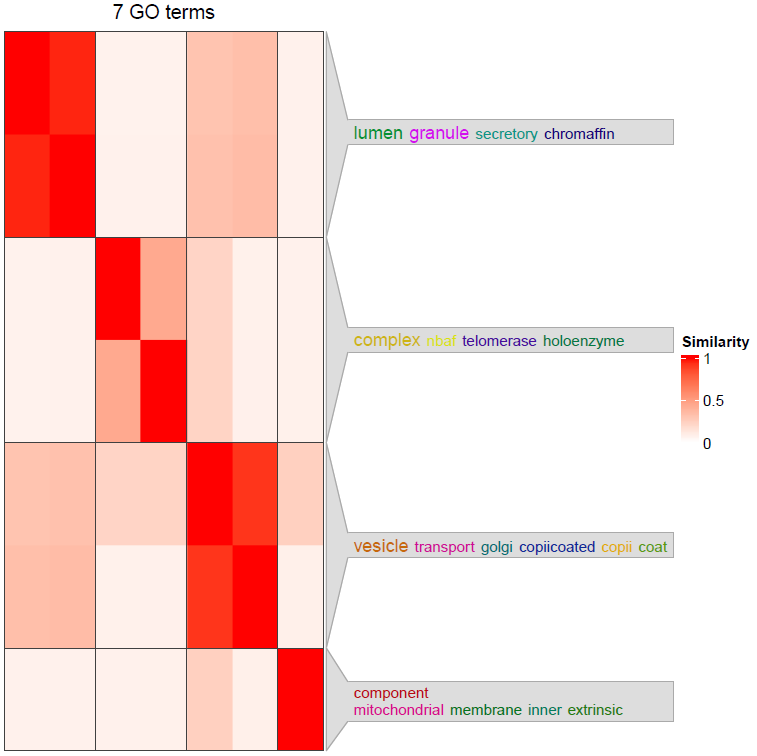


**Figure S26**: The heatmap corresponding to the 7 over-represented GO-terms cellular component for the comparison between DO and CBX after 22 hours of maturation, with a color gradient from white to red for similarity between 0 – 1.


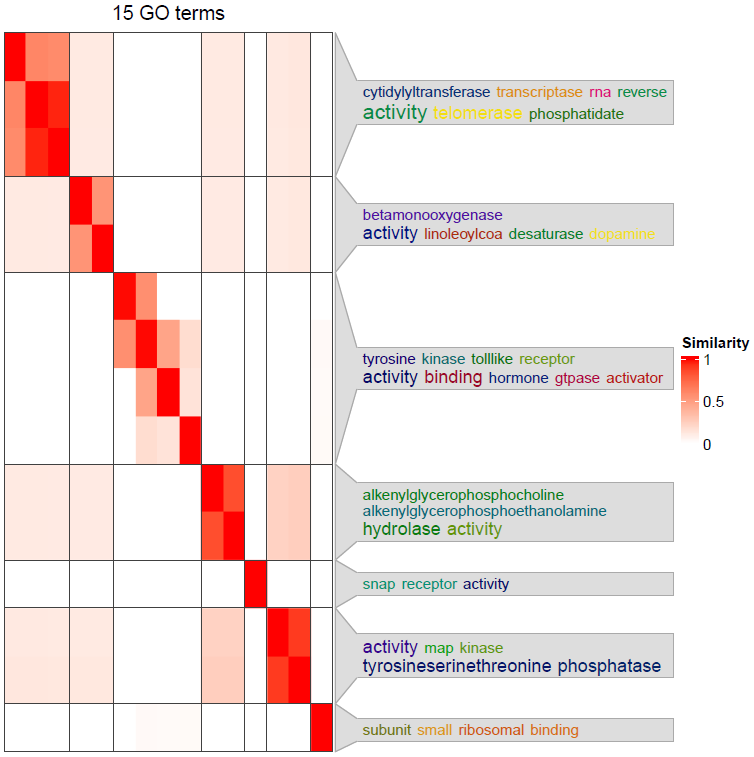


**Figure S27**: The heatmap corresponding to the 15 over-represented GO-terms molecular function for the comparison between DO and CBX after 22 hours of maturation, with a color gradient from white to red for similarity between 0 – 1.


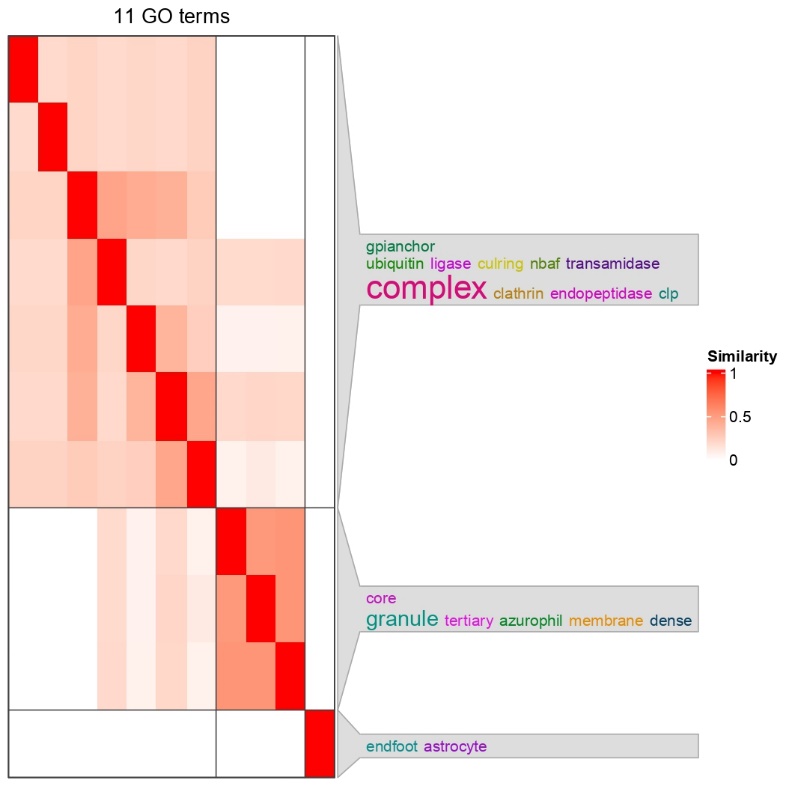


Figure S28: The heatmap corresponding to the 11 over-represented GO-terms from cellular component for the comparison between control and CBX after 22 hours of maturation, white a color gradient from with to red for similarity between 0 – 1.


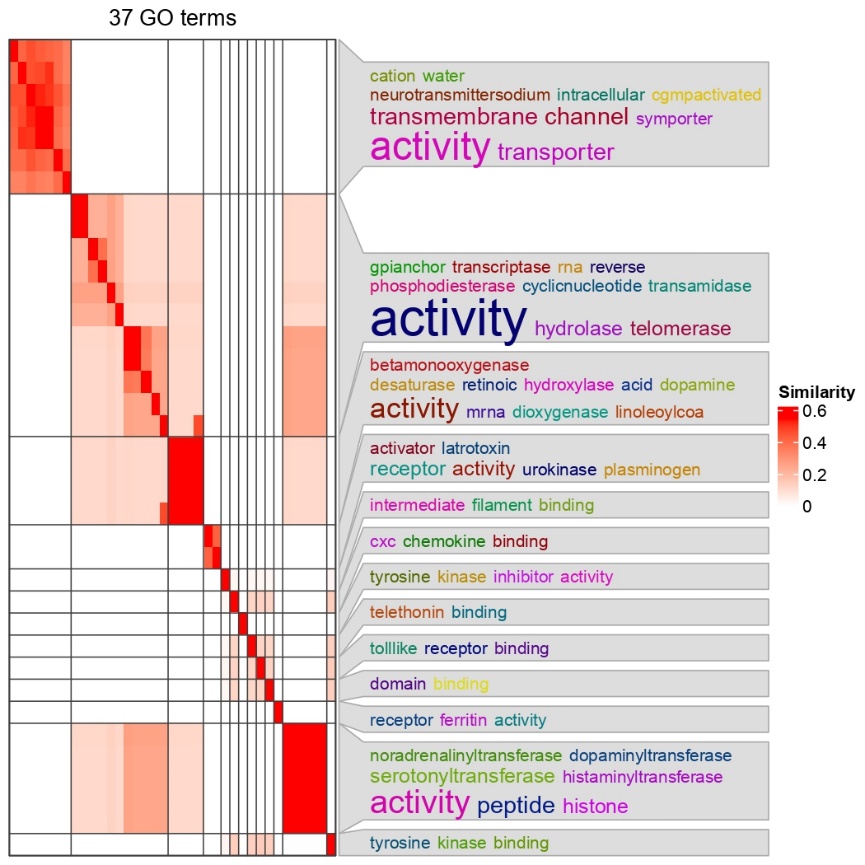


Figure S29: The heatmap corresponding to the 37 over-represented GO-terms from molecular function for the comparison between control and CBX after 22 hours of maturation, with a color gradient from white to red for similarity between 0 – 0.6.
